# Sesamin as a Context-Dependent Redox Modulator: Interactions with Organelle Quality Control and Regulated Cell Death

**DOI:** 10.3390/antiox15101299

**Published:** 2026-10-08

**Authors:** I-Ta Lee, Yu-Chia Hsu, Yung-Li Wang, Sheng-Wei Feng, Thi Thuy Tien Vo, Tong-Hsien Chow

**Affiliations:** 1 School of Dentistry, College of Oral Medicine, Taipei Medical University, Taipei 110301, Taiwan; cetuswang@tmu.edu.tw (Y.-L.W.); shengwei@tmu.edu.tw (S.-W.F.); 2 Department of Agronomy, National Chiayi University, Chiayi 600355, Taiwan; hsuychia@mail.ncyu.edu.tw; 3 Faculty of Dentistry, Nguyen Tat Thanh University, Ho Chi Minh City 70000, Vietnam; vtttien@ntt.edu.vn; 4 Department of Sports Science, R.O.C. Military Academy, Kaohsiung 830208, Taiwan; thchowma@staff.mil.tw

**Keywords:** sesamin, redox regulation, bioavailability, pharmacokinetics, regulated cell death

## Abstract

Sesamin is a furofuran lignan in sesame with reported antioxidant, anti-inflammatory, metabolic, and anticancer activities. However, its biological effects vary according to exposure, metabolism, cellular state, and stress-response capacity. This review examines sesamin as a context-dependent redox modulator, focusing on bioavailability, metabolite-dependent activity, organelle quality control, and regulated cell death. We integrate evidence on cytochrome P450-mediated metabolism, catechol formation, conjugation, tissue availability, mitochondrial redox regulation, mitophagy, autophagic flux, lysosomal function, innate immune signalling, and their intersections with apoptosis, ferroptosis, and pyroptosis. In non-malignant injury models, sesamin generally attenuates oxidative damage, preserves organelle function, and limits inflammatory or ferroptotic injury, whereas higher concentrations in selected malignant-cell models may increase oxidative stress, disrupt survival signalling, or enhance susceptibility to cell death. These divergent outcomes appear to depend on molecular species, dose, metabolic competence, baseline redox pressure, and organelle reserve. Translational limitations include sparse human pharmacokinetic and efficacy data, uncertain tissue exposure, frequent use of pharmacologically unverified micromolar concentrations, and limited metabolite-specific testing. Future studies should therefore combine exposure-matched experimental systems with quantitative redox measurements, organelle-flux assays, and pathway-specific validation to define the biological relevance of sesamin-mediated redox regulation and cell-fate control.

## 1. Introduction

Redox homeostasis is governed not simply by the bulk abundance of reactive oxygen species (ROS), but by the chemical identity of the oxidant, its site and duration of generation, the molecular targets engaged, and the capacity of local reducing systems [1,2]. Low, transient, and spatially restricted oxidant fluxes participate in physiological redox signalling, whereas excessive or unresolved oxidant production causes oxidative distress and macromolecular damage [2]. Accordingly, global measurements of ROS or oxidative damage should not be interpreted in isolation as evidence for a defined redox mechanism. Current methodological guidance instead recommends chemically appropriate probes, orthogonal validation, and consideration of the relevant subcellular compartment and temporal window [1,3]. The disappointing clinical performance of many nonspecific antioxidants further indicates that therapeutic activity cannot be predicted solely from radical-scavenging capacity measured in cell-free systems [4]. Redox regulation also intersects with metabolic adaptation, organelle quality control, and multiple regulated cell death (RCD) pathways, making cellular outcome dependent on the biological context in which an oxidant-modulating intervention is applied [1,5,6].

Sesamin is an oil-soluble furofuran lignan occurring predominantly in sesame (*Sesamum indicum* L.), particularly in sesame seeds and seed-derived oil. It is one of the major naturally occurring sesame lignans, together with structurally related compounds such as sesamolin, and its abundance can vary according to cultivar, seed composition, and processing conditions [7]. Sesame seeds and sesame oil therefore represent the principal dietary sources of sesamin. Experimental studies have attributed antioxidant, anti-inflammatory, metabolic, cardiovascular, neuroprotective, hepatoprotective, and anticancer activities to sesamin [7]. Previous reviews have summarised its general pharmacology, anticancer properties, cardiovascular effects, and immunomodulatory and anti-inflammatory activities [7,8,9,10,11]. However, these syntheses were largely organised by disease or pharmacological outcome, with changes in ROS, lipid peroxidation, antioxidant enzymes, inflammatory signalling, autophagy, and apoptosis often treated as parallel observations rather than as components of an integrated redox–organelle–cell-death network. Most also preceded the 2024–2026 expansion of mechanistic studies addressing mitophagy, autophagic flux, mitochondrial deoxyribonucleic acid (mtDNA) stress, pyroptosis, and ferroptosis.

The conventional description of sesamin as a direct antioxidant is complicated by its metabolism and systemic exposure. In a cell-free metabolic system, unmetabolised sesamin lacked detectable antioxidant activity, whereas catechol metabolites generated in rat liver homogenates exhibited strong radical-scavenging activity [12]. Importantly, the absence of detectable antioxidant activity under these cell-free conditions should not be interpreted as indicating that parent sesamin is biologically inactive. Parent sesamin can exert cellular effects independently of hepatic catechol formation, including modulation of intracellular signalling, mitochondrial function, autophagy-associated responses, and cell-death pathways, as demonstrated in several in vitro models discussed below. The sesamin-derived monocatechol metabolite SC1 was subsequently shown to interact with annexin A1 and to exert annexin A1-dependent anti-inflammatory effects [13]. Monocatechol metabolites of sesamin and episesamin also increased autophagic flux more strongly than their parent compounds by selectively inhibiting mechanistic target of rapamycin complex 1 (mTORC1) and reducing inhibitory phosphorylation of Unc-51-like autophagy-activating kinase 1 (ULK1) and transcription factor EB (TFEB) [14]. Human pharmacokinetic data demonstrated the appearance of parent lignans and monocatechol metabolites after oral administration, but the reported peak plasma concentration of parent sesamin was only approximately 7.6 nM [15]. Exposure is also relevant to interaction assessment because sesamin inhibited cytochrome P450 2C9 (CYP2C9)-dependent *S*-warfarin 7-hydroxylation in vitro, although the clinical relevance of this finding remains uncertain [16]. Biological exposure should therefore be understood as a time-dependent combination of parent sesamin, its metabolites, tissue distribution, and local metabolic competence.

Recent studies have connected sesamin with several components of organelle quality control. Reported mechanisms include mitophagy associated with phosphatase and tensin homolog (PTEN)-induced kinase 1 (PINK1) and Parkin [17], autophagic flux regulated by mTORC1 [14], redox protection involving sirtuin 5 (SIRT5)/forkhead box O3a (FOXO3a) signalling [18], and suppression of cyclic GMP–AMP synthase (cGAS)–stimulator of interferon genes (STING) signalling [19]. These findings identify mitochondrial turnover, lysosomal function, nutrient sensing, and mtDNA surveillance as candidate nodes of sesamin action, but they do not establish a single linear mechanism. The observation that sesamin enhanced autophagic flux in one setting but attenuated stress-associated autophagy in another further suggests that the direction of the response depends on cellular context [14,18].

Evidence linking sesamin to RCD has also expanded. Sesamin suppressed hepatocyte pyroptosis through a protein kinase Cδ (PKCδ)/NLR family CARD domain-containing protein 4 (NLRC4)/caspase-1 pathway in experimental nonalcoholic steatohepatitis [20]. Ferroptosis-related protection has been associated with forkhead box O1 (FOXO1) regulation in toxic liver injury [21], a methyltransferase-like 3 (METTL3)–N^6^-methyladenosine (m^6^A)–sterol regulatory element-binding transcription factor 1 (SREBF1) axis in endothelial injury [22], and nuclear factor erythroid 2-related factor 2 (NRF2)/solute carrier family 7 member 11 (SLC7A11)/glutathione peroxidase 4 (GPX4) signalling in ischaemic skin flaps [23]. These studies support modulation of pyroptosis-, ferroptosis-, and apoptosis-related processes, but changes in ROS, iron, GPX4, SLC7A11, lipid peroxidation, or viability alone do not establish a specific RCD mechanism. Functional rescue, genetic perturbation, and complementary biochemical endpoints remain necessary [6,24].

A central unresolved question is why sesamin is cytoprotective in non-malignant metabolic, toxic, inflammatory, and ischaemic models [17,18,19,20,21,22,23] yet promotes or sensitises cell death in selected tumour models. Reported effects of parent sesamin include autophagy-associated apoptosis and pyroptosis in murine T-cell lymphoma [25], p53/PTEN-associated apoptosis in cervical cancer cells [26], and increased chemosensitivity in lung adenocarcinoma cells [27]. However, these studies used micromolar parent-sesamin concentrations that substantially exceeded reported human parent-compound exposure [15,26,27]. They therefore provide mechanistic observations with limited direct pharmacological translatability.

These apparently opposing outcomes motivate the context-dependent hypothesis developed in Section 5. Direct comparisons of normal and malignant cells under exposure-matched and metabolite-informed conditions remain scarce; therefore, context-dependent cytoprotection versus cytotoxicity should be treated as a testable hypothesis rather than an established universal property of sesamin.

**Figure 1:**
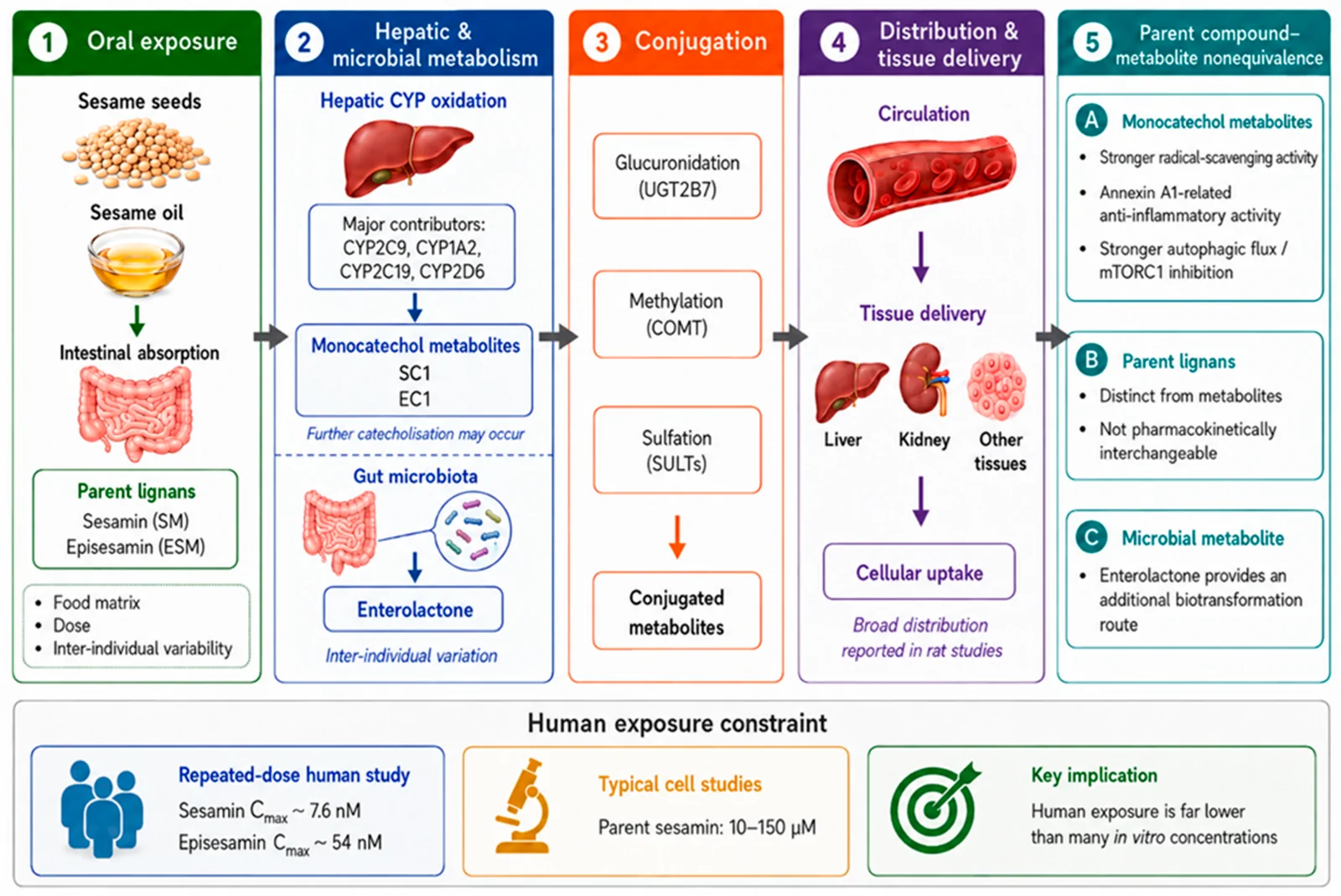
Oral exposure, metabolism, distribution, and parent compound–metabolite nonequivalence of sesamin. Dietary sesamin (SM) and episesamin (ESM) are absorbed from sesame-containing foods and undergo hepatic cytochrome P450 (CYP)-mediated oxidation to monocatechol metabolites, including SC1 (sesamin monocatechol) and EC1 (episesamin monocatechol). CYP2C9, CYP1A2, CYP2C19, and CYP2D6 contribute to monocatechol formation. Further catecholisation and phase II conjugation through glucuronidation, predominantly involving uridine diphosphate-glucuronosyltransferase 2B7 (UGT2B7), methylation by catechol-*O*-methyltransferase (COMT), and sulfation by sulfotransferases (SULTs) generate additional metabolites. Intestinal microbiota can also convert sesamin to enterolactone, with substantial interindividual variability. Sesamin-derived compounds are distributed to peripheral tissues, with broad distribution demonstrated primarily in animal studies. Parent lignans and their metabolites are not pharmacologically equivalent: monocatechol metabolites can exhibit stronger radical-scavenging activity, annexin A1-related anti-inflammatory effects, and greater stimulation of autophagic flux through inhibition of mechanistic target of rapamycin complex 1 (mTORC1) than the parent compounds. Human pharmacokinetic data indicate low circulating parent-lignan exposure, with reported maximum plasma concentrations (*C*_max_) of approximately 7.6 nM for sesamin and 54 nM for episesamin, whereas mechanistic cell studies frequently use parent sesamin in the micromolar range. These exposure differences should be considered when interpreting the translational relevance of in vitro findings. The scientific concepts, mechanistic relationships, and intended content were determined by the authors. ChatGPT (2026) image-generation tools (OpenAI) were used to assist with the visual rendering and refinement of the schematic illustration based on detailed author-provided instructions and prompts. All graphical elements were subsequently reviewed and approved by the authors. This figure is an original schematic created specifically for this review and is not reproduced or adapted from previously published material.

**Figure 2:**
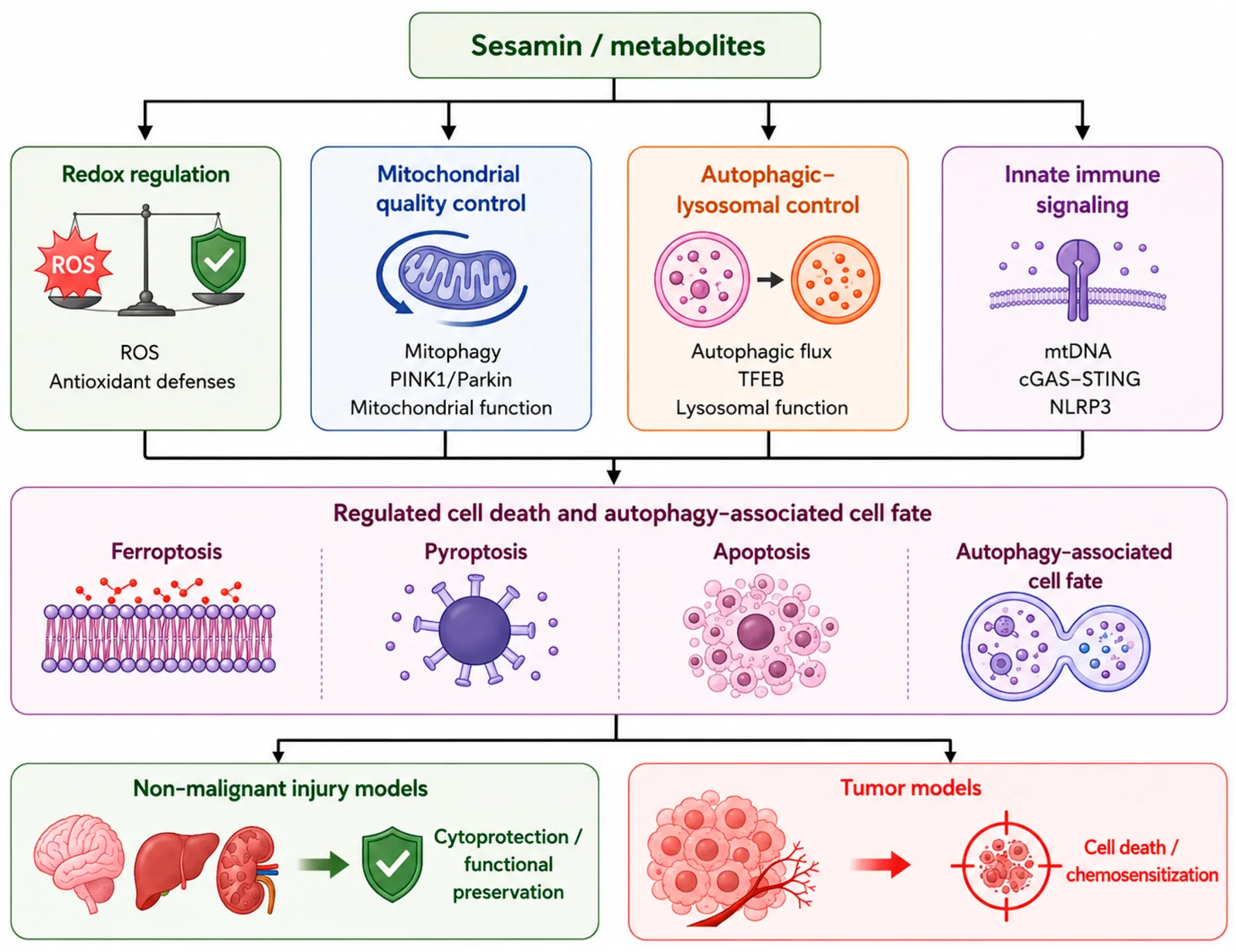
Proposed intersections among redox regulation, organelle quality control, innate immune signalling, and regulated cell death in response to sesamin and its metabolites. Sesamin and its metabolites may influence four interconnected mechanistic domains: redox regulation, mitochondrial quality control, autophagic–lysosomal control, and innate immune signalling. Redox regulation involves changes in reactive oxygen species (ROS) and antioxidant defences, whereas mitochondrial quality control includes phosphatase and tensin homolog-induced kinase 1 (PINK1)/Parkin-associated mitophagy and preservation of mitochondrial function. Autophagic–lysosomal control involves autophagic flux, transcription factor EB (TFEB), and lysosomal function, while mitochondrial stress may engage mitochondrial DNA (mtDNA)-dependent cyclic GMP–AMP synthase–stimulator of interferon genes (cGAS–STING) signalling and NOD-like receptor family pyrin domain-containing 3 (NLRP3)-related innate immune responses. These processes intersect with ferroptosis, pyroptosis, apoptosis, and autophagy-associated cell fate. Across non-malignant injury models, sesamin is generally associated with cytoprotection and functional preservation, whereas in selected tumour models it may promote regulated cell death or enhance chemosensitivity. The scheme is intended as an integrative, context-dependent framework rather than a single linear or universally established causal pathway. The scientific concepts, mechanistic relationships, and intended content were determined by the authors. ChatGPT image-generation tools (OpenAI) were used to assist with the visual rendering and refinement of the schematic illustration based on detailed author-provided instructions and prompts. All graphical elements were subsequently reviewed and approved by the authors. This figure is an original schematic created specifically for this review and is not reproduced or adapted from previously published material.

**Figure 3:**
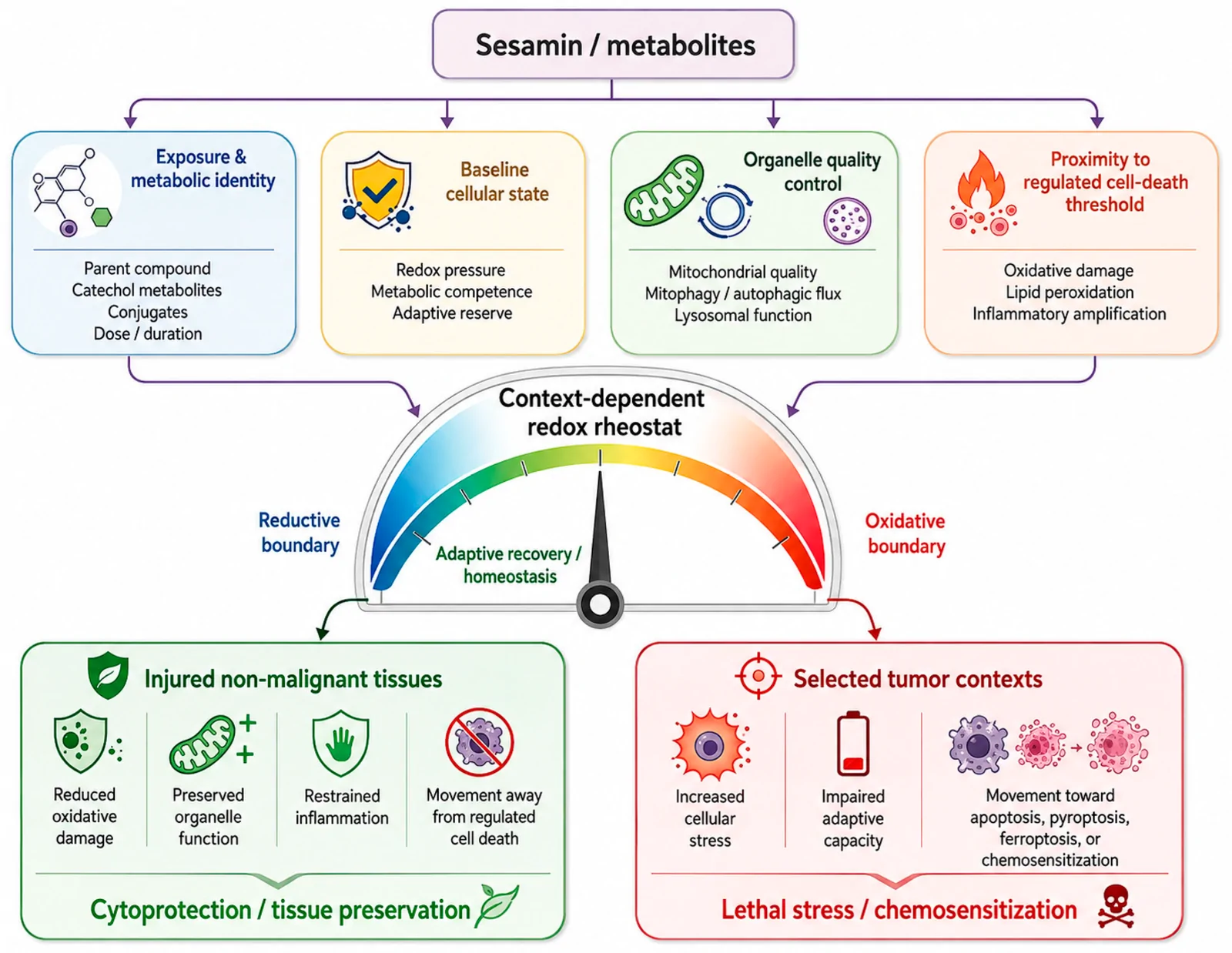
Context-dependent redox-rheostat framework for sesamin and its metabolites. The proposed rheostat model integrates molecular exposure and metabolic identity, baseline cellular state, organelle quality control, and proximity to a regulated cell-death threshold as major determinants of the biological response to sesamin and its metabolites. Parent sesamin, catechol metabolites, conjugated derivatives, dose, and exposure duration may provide distinct molecular inputs, whereas baseline redox pressure, metabolic competence, and adaptive reserve influence cellular susceptibility. Mitochondrial quality control, mitophagy, autophagic flux, and lysosomal function represent potential inflection points that may determine whether redox perturbation is resolved adaptively or progresses toward regulated cell death. At the oxidative boundary, sustained oxidative damage, lipid peroxidation, mitochondrial dysfunction, and inflammatory amplification may exceed cellular repair and buffering capacity and promote apoptosis, pyroptosis, or ferroptosis. In injured non-malignant tissues, sesamin-associated responses generally shift cells toward adaptive recovery, organelle preservation, restrained inflammation, and cytoprotection. In selected tumour contexts, increased cellular stress or impaired adaptive capacity may instead move cells toward regulated cell death or chemosensitisation. The opposite reductive boundary is included as a conceptual constraint on physiological redox signalling but has not been demonstrated experimentally for sesamin. The rheostat is therefore an integrative, hypothesis-generating framework rather than a universal quantitative dose–response relationship. The scientific concepts, mechanistic relationships, and intended content were determined by the authors. ChatGPT image-generation tools (OpenAI) were used to assist with the visual rendering and refinement of the schematic illustration based on detailed author-provided instructions and prompts. All graphical elements were subsequently reviewed and approved by the authors. This figure is an original schematic created specifically for this review and is not reproduced or adapted from previously published material.

In this review, we examine sesamin as a context-dependent redox modulator and use a redox-rheostat framework to integrate its metabolism, organelle quality control, and regulated cell-death responses. The term “rheostat” denotes an integrative model rather than a single molecular switch or direct target. We examine whether sesamin and its metabolites shift cells between adaptive redox recovery and RCD through mitochondrial quality control, autophagic flux, inflammatory organelle signalling, and susceptibility to lipid peroxidation. We first evaluate exposure, metabolism, and pharmacologically relevant concentrations, then integrate evidence concerning redox regulation, organelle quality control, ferroptosis, pyroptosis, apoptosis, and autophagy-associated cell fate. Finally, we assess translational limitations and propose experimental standards for distinguishing causal redox mechanisms from correlative antioxidant endpoints. Figure 1, Figure 2 and Figure 3 provide complementary visual summaries of sesamin exposure and metabolism, the intersections among redox regulation, organelle quality control and regulated cell death, and the proposed context-dependent redox-rheostat framework, respectively.

## 2. Exposure and Metabolism

The nutritional and biological interpretation of sesamin requires consideration of its bioavailability and a clear distinction between administered dose, circulating parent compound, metabolite exposure, and concentrations achieved within target tissues. Available evidence indicates that systemic and tissue exposure may comprise a time-dependent mixture of parent sesamin, episesamin, catechol metabolites, conjugated derivatives, and microbiota-derived enterolignans [15,28,29,30]. These molecular species should not be assumed to have equivalent redox, signalling, or pharmacological properties.

### 2.1. Human Exposure and Pharmacokinetic Constraints

The principal repeated-dose human pharmacokinetic study did not administer sesamin alone. In a single-blind, placebo-controlled, parallel-group study, 48 healthy participants were randomly assigned to receive either placebo or 50 mg of a sesame-lignan preparation containing sesamin and episesamin at a 1:1 ratio once daily for 28 days [15]. Although 24 participants received the lignan preparation, pharmacokinetic analyses were conducted in only 10 of these participants [15]. Sesamin, episesamin, SC1, and the episesamin-derived monocatechol metabolite EC1 reached maximum plasma concentrations at approximately 5 h. SC1 and EC1 subsequently declined with terminal half-lives of 2.4 and 3.4 h, respectively, whereas the terminal half-life of episesamin was 7.1 h [15]. The reported maximum plasma concentrations (*C*_max_) of parent sesamin and episesamin were approximately 7.6 and 54 nM, respectively [15]. Plasma concentrations of the parent lignans reached a steady state by day 7, with no evidence of accumulation during repeated administration [15]. These results provide useful exposure boundaries but were obtained from a small pharmacokinetic subset and do not establish tissue concentrations or clinical efficacy. Importantly, experimentally determined plasma protein binding and unbound fractions of sesamin have not been reported in humans. Available computational estimates of plasma protein binding or fraction unbound should not be interpreted as measured pharmacokinetic values. Consequently, the reported total plasma concentration of parent sesamin cannot be assumed to represent its unbound systemic exposure or the freely available concentration within target tissues, and comparisons with nominal concentrations used in cell culture should therefore be interpreted cautiously.

A separate human study examined urinary elimination after six healthy volunteers consumed sesame oil containing 508 μmol (approximately 180 mg) of sesamin [28]. Urinary recovery of a sesamin monocatechol metabolite ranged from 22.2% to 38.6% of the ingested dose, with a mean of 29.3 ± 5.6%, and occurred predominantly during the first 12 h [28]. Because the urine samples were treated with β-glucuronidase and sulfatase before analysis, the reported recovery represented the total catechol metabolite measurable after deconjugation and did not distinguish the free urinary metabolite from its glucuronide or sulfate conjugates [28]. This distinction is important because hydrolysis before measurement can conceal the relative abundance of individual circulating or excreted conjugates.

Intestinal microbial metabolism provides an additional route of biotransformation. In a preliminary study involving four participants, the appearance of mammalian lignans in plasma after a single serving of sesame seeds indicated that sesamin was a precursor of enterolactone [29]. Incubation of sesamin with human faecal microbiota also generated enterolactone as the principal end product and identified three metabolic intermediates [29]. However, substantial interindividual variation was observed, and the very small sample precludes quantitative generalisation to broader populations [29]. The extent to which enterolactone or other microbiota-derived metabolites contribute to the biological effects attributed to orally administered sesamin remains unclear. In the animal and human studies reviewed here, these metabolites were generally not quantified together with mechanistic or clinical endpoints. Consequently, observed in vivo effects cannot be assigned specifically to parent sesamin, hepatic metabolites, or microbiota-derived products. Interindividual variation in gut microbial metabolism may therefore represent an additional source of variability in sesamin response, and future studies should measure enterolactone and related metabolites alongside parent lignans and hepatic metabolites when evaluating systemic effects.

These exposure data are essential for interpreting cell-culture experiments. Parent-sesamin concentrations of 75–150 μM were used in the cervical cancer study, whereas concentrations up to 100 μM were examined in the lung adenocarcinoma study [26,27]. These concentrations substantially exceed the nanomolar plasma concentrations of parent sesamin reported after oral administration in humans [15]. However, plasma exposure alone does not exclude the possibility of higher or prolonged exposure within specific tissues. In rats, sesamin-derived radioactivity was widely distributed and was particularly prominent in the liver and kidney, although the material detected in these organs consisted predominantly of conjugated metabolites rather than unchanged parent sesamin [30]. Thus, local tissue exposure may not be directly reflected by circulating parent-sesamin concentrations. Nevertheless, quantitative human tissue concentrations of parent sesamin and its major metabolites remain unavailable, and there is currently no evidence that micromolar concentrations of unchanged sesamin comparable to those used in many cell-culture studies are achieved in human target tissues. High-concentration in vitro experiments should therefore be regarded as mechanistically informative, while their physiological relevance requires confirmation by tissue-level exposure measurements.

### 2.2. Sequential Hepatic Metabolism and Stereochemical Dependence

Sesamin undergoes sequential hepatic oxidation and conjugation rather than remaining exclusively as the unchanged parent compound. Studies using recombinant CYP enzymes and human liver microsomes showed that several CYP isoforms can catalyse monocatechol formation [31]. Although recombinant CYP2C19 exhibited the highest catalytic efficiency, adjustment for the estimated abundance of individual CYP isoforms in human liver produced the following predicted contribution order: CYP2C9, CYP1A2, CYP2C19, and CYP2D6 [31]. Correlation and antibody-inhibition analyses using human liver microsomes further identified CYP2C9 as the principal contributor to sesamin monocatecholisation, with CYP1A2 making a secondary contribution [31].

The resulting sesamin monocatechol can undergo additional catecholisation, glucuronidation, or methylation [32]. In human liver microsomes, glucuronidation of the monocatechol was substantially greater than conversion to the dicatechol, whereas further catecholisation predominated in rat liver microsomes [32]. Experiments with recombinant human uridine diphosphate-glucuronosyltransferases (UGTs) and microsomes from 10 human livers identified UGT2B7 as the principal isoform associated with monocatechol glucuronidation [32]. Catechol-*O*-methyltransferase (COMT)-dependent methylation was also detected in human liver cytosol [32]. These results demonstrate a marked species difference and caution against directly extrapolating the relative abundance or antioxidant activity of rat sesamin metabolites to humans.

Metabolism is also stereochemically dependent. Episesamin was converted into *S*- and *R*-monocatechol epimers in human liver microsomes, with CYP2C9 primarily responsible for formation of the *S*-monocatechol and CYP1A2 responsible for the *R*-monocatechol [33]. CYP2C9 contributed much more strongly than CYP1A2 to sesamin metabolism, whereas the contributions of CYP2C9 and CYP1A2 to episesamin metabolism were approximately equivalent [33]. The monocatechol epimers of episesamin were subsequently converted into dicatechol, glucuronide, and methylated metabolites, but the relative contributions of these pathways differed from those observed for sesamin [33]. Therefore, sesamin and episesamin should not be treated as pharmacokinetically interchangeable, even when administered together in an approximately 1:1 mixture.

### 2.3. Conjugation, Tissue Delivery, and Metabolite-Dependent Activity

A mass-balance study using a single oral dose of 5 mg/kg [^14^C]sesamin in rats found that plasma radioactivity peaked at 1 h and declined with a terminal half-life of 4.7 h [30]. Cumulative radioactivity recovery was 37.5 ± 3.1% in urine and 58.7 ± 4.8% in faeces. In bile-duct-cannulated rats, 66.3 ± 8.4% of administered radioactivity was recovered in bile and 27.8 ± 10.2% in urine [30]. Radioactivity was widely distributed and was particularly prominent in the liver and kidney, where sesamin-related material was present mainly as conjugated metabolites [30]. These values describe total sesamin-derived radioactivity and must not be interpreted as the distribution or excretion of unchanged sesamin.

Direct analysis without enzymatic deconjugation identified two sulfate conjugates of SC1 in the liver and plasma of rats administered sesamin at 100 mg/kg [34]. Their *C*_max_ values were approximately fivefold and tenfold higher than that of parent sesamin [34]. The catalytic efficiency of sulfate conjugation in human liver cytosol was 1.7-fold that measured in rat liver cytosol, and recombinant human sulfotransferases were capable of forming these conjugates [34]. These in vitro results support the plausibility of hepatic sulfate formation in humans, but direct in vivo confirmation of the relative abundance of individual sulfate conjugates in human plasma remains unavailable.

Conjugation does not necessarily indicate irreversible biological inactivation. In mouse macrophage-like J774.1 cells, the inhibitory effects of SC1 glucuronides on lipopolysaccharide-induced nitric oxide production increased with incubation time and were associated with intracellular accumulation of unconjugated SC1 and its methylated derivative [35]. The effects of the glucuronides were abolished by β-glucuronidase inhibition and enhanced by COMT inhibition, supporting cellular deconjugation followed by intracellular metabolism in this model [35]. SC1 and its glucuronides also suppressed interferon-β (IFN-β) and inducible nitric oxide synthase (iNOS) expression [35]. These findings support a cell-specific deconjugation mechanism in J774.1 cells; they do not establish that glucuronides universally function as active delivery reservoirs in human tissues.

Parent compound–metabolite nonequivalence is also evident in functional studies. Catechol metabolites showed greater radical-scavenging activity than unmetabolised sesamin under the cell-free conditions examined [12]. SC1 interacted with annexin A1 and exerted annexin A1-dependent anti-inflammatory effects in cellular and animal experiments [13]. More recently, monocatechol metabolites of sesamin and episesamin enhanced autophagic flux more strongly than their parent compounds through selective inhibition of mTORC1 [14]. Collectively, these findings indicate that the biological activity assigned to orally administered sesamin may arise from parent sesamin, one or more metabolites, or their sequential interconversion, depending on the experimental system. Whether the major sesamin metabolites and their conjugates can effectively cross the mammalian blood–brain barrier remains uncertain. Direct quantitative evidence for blood–brain barrier penetration of the major catechol metabolites, including SC1 and EC1, or their glucuronide and sulfate conjugates is currently lacking. Given their greater polarity relative to parent sesamin, central nervous system exposure to these metabolites and conjugates should not be inferred from peripheral plasma concentrations. Dedicated studies quantifying individual sesamin-derived molecular species in plasma, cerebrospinal fluid, and brain tissue are therefore required before their blood–brain barrier permeability and central nervous system bioavailability can be established.

Formulation can further alter exposure. In a 2026 rat study, oral administration of a slowly self-emulsifying sesamin formulation at 20 mg/kg produced a 7.9-fold greater plasma area under the concentration–time curve than crystalline sesamin [36]. Relative to a conventional self-emulsifying formulation, the slowly self-emulsifying formulation reduced *C*_max_ by 22% and prolonged the mean residence time to 5.3 h [36]. This study demonstrates that oral sesamin exposure can be modified substantially by formulation, but it does not establish corresponding efficacy, safety, or pharmacokinetic improvement in humans. The principal pathways linking oral exposure, hepatic and microbial metabolism, conjugation, tissue delivery and parent compound–metabolite nonequivalence are illustrated in Figure 1.

### 2.4. Food–Drug Interaction Potential and Exposure-Informed Interpretation

Because CYP2C9 participates in sesamin monocatechol formation, sesamin has also been examined as a potential inhibitor of CYP2C-mediated drug metabolism. Early human liver microsomal experiments detected mechanism-based inhibition of CYP2C9 by sesamin [31]. The resulting metabolic–intermediate complex was subsequently found to be unstable, with the associated spectral signal disappearing within 30 min after metabolism was terminated [37]. In rats pretreated with sesamin at 10 or 100 mg/kg for 3 days, no significant differences were detected in the maximum plasma concentration (*C*_max_), time to maximum plasma concentration (*T*_max_), or area under the concentration–time curve of diclofenac [37]. Seven-day sesamin administration also did not alter hepatic diclofenac hydroxylation activity in rats [37]. These findings demonstrate an absence of a measurable sesamin–diclofenac interaction under the conditions of that rat study, but they cannot establish the absence of an interaction in humans.

Two human liver microsomal studies produced markedly different estimates of sesamin-mediated inhibition of warfarin 7-hydroxylation. The first reported non-competitive inhibition with a *K*_i_ of 202 ± 18 nM [38]. A subsequent study reported competitive inhibition of *S*-warfarin 7-hydroxylation, with *K*_i_ values of 15.7 μM in human liver microsomes and 13.1 μM using recombinant CYP2C9 [16]. The human liver microsomal *K*_i_ values reported in the two studies differed by approximately 78-fold [16,38]. The reason for this discrepancy remains unresolved, although differences in assay design and kinetic interpretation may have contributed. Consequently, neither value should be used alone to predict a clinical interaction. Direct comparison of these in vitro *K*_i_ values with human plasma exposure also requires caution. The reported parent-sesamin *C*_max_ of approximately 7.6 nM is below both published human liver microsomal *K*_i_ estimates, but total plasma concentration does not represent the unbound concentration available to hepatic CYP enzymes, and experimentally determined human protein binding is unavailable. In addition, hepatic exposure may differ from systemic plasma exposure, metabolites may contribute differently to enzyme inhibition, and the reported *K*_i_ values were obtained under different experimental conditions. These uncertainties preclude reliable prediction of the magnitude of a clinical interaction from the available in vitro data alone.

A 2026 study using recombinant human drug-metabolising enzymes tested sesamin at 0, 1, 10, and 100 μM [39]. No significant inhibition was detected across the examined UGT isoforms, whereas CYP2C9 and CYP2C19 activities were inhibited [39]. Interaction-risk calculations particularly highlighted phenytoin, a CYP2C9 substrate with a narrow therapeutic index [39]. These findings establish in vitro inhibitory potential and a model-based interaction prediction, but they do not demonstrate a clinically observed food–drug interaction in humans.

Overall, the existing evidence supports metabolism-dependent interaction potential but remains insufficient to define clinical risk. None of the cited studies directly measured a sesamin-related pharmacokinetic interaction in patients receiving warfarin, phenytoin, or another narrow-therapeutic-index CYP2C9 or CYP2C19 substrate. Until controlled human interaction studies are available, dietary sesame exposure, concentrated sesamin supplementation, and high-dose experimental formulations should not be assumed to produce equivalent interaction risks. Exposure-informed experiments should measure parent sesamin and its major unconjugated and conjugated metabolites, report unbound concentrations where feasible, and compare these values with concentrations required for CYP inhibition or redox-related biological effects. The principal pharmacokinetic, metabolic, and interaction evidence for sesamin, episesamin, and their metabolites is summarised in Table 1.

## 3. Redox and Organelle Quality Control

Framing sesamin simply as an antioxidant obscures the dynamic nature of redox regulation. ROS are not only mediators of molecular damage but also spatially restricted signals that regulate metabolism, stress adaptation, organelle turnover, and cell fate [1,2]. Accordingly, a reduction in a nonspecific oxidation-sensitive fluorescent signal, malondialdehyde (MDA) concentration, or a single antioxidant-enzyme measurement does not by itself demonstrate direct radical scavenging or identify the reactive species and subcellular source involved [3,4]. The evidence for sesamin should therefore be separated into three mechanistic levels: direct redox chemistry, adaptive redox signalling, and preservation or removal of damaged organelles.

### 3.1. From Radical Scavenging to Adaptive Redox Signalling

The direct redox chemistry of sesamin is metabolite-dependent. In the in vitro systems used by Nakai et al., parent sesamin showed no detectable antioxidative activity, whereas its enzymatically generated mono- and dicatechol metabolites displayed strong radical-scavenging activity [12]. This finding establishes chemical plausibility for metabolite-mediated antioxidant activity but does not demonstrate that the same reactions dominate within intact cells, where local concentration, conjugation, membrane partitioning, and competition with endogenous redox systems determine biological effects.

Evidence for transcriptional redox adaptation has recently been obtained in a non-mammalian model. In *Drosophila* larvae carrying an antioxidant response element (ARE)–green fluorescent protein (GFP) reporter, dietary sesamin activated Cap ‘n’ collar isoform C, the fly orthologue of mammalian NRF2, in the central nervous system (CNS), gut, and salivary glands [40]. Ectopic expression of *Keap1* in glial cells attenuated sesamin-induced reporter activation in the CNS more strongly than neuronal *Keap1* expression. Sesamin also increased the transcripts of the NRF2-responsive cytochrome P450 genes *Cyp6a2* and *Cyp6g1*, although this transcriptional response did not confer resistance to imidacloprid [40]. These genetic data support NRF2-dependent signalling in *Drosophila* glia but cannot establish NRF2 target engagement in mammalian tissues or humans.

In mammalian cells, metabolite-dependent evidence was provided by SC1. In PC12 cells, the monocatechol metabolites SC1 and EC1 activated ARE signalling more strongly than their parent compounds, while SC1 promoted NRF2 nuclear translocation and heme oxygenase-1 (HO-1) expression [41]. N-acetyl-L-cysteine (NAC) abolished SC1-induced p38 mitogen-activated protein kinase (p38 MAPK) phosphorylation and HO-1 induction, whereas zinc protoporphyrin and dominant-negative NRF2 diminished its protection against hydrogen peroxide (H_2_O_2_)-induced cell death [41]. These interventions support metabolite-dependent NRF2/ARE–HO-1 signalling, although the findings were obtained in a rat pheochromocytoma cell line and do not establish target engagement in humans.

A separate 2026 study identified SIRT5 and FOXO3a as potential mediators of redox protection in diquat-induced acute kidney injury (AKI) [18]. In human proximal tubular HK-2 cells, diquat increased ROS generation, mitochondrial damage, apoptosis, and autophagy-associated responses. Sesamin attenuated these changes and reduced renal injury in mice [18]. *SIRT5* knockdown aggravated diquat toxicity, whereas *SIRT5* overexpression was protective. Molecular docking and a cellular thermal shift assay (CETSA) were reported to indicate an interaction between sesamin and SIRT5, with docking identifying Phe70 as a possible interaction site [18]. The study linked the protective phenotype to SIRT5/FOXO3a signalling; however, these approaches do not independently establish binding affinity, structural interaction, or in vivo target occupancy.

### 3.2. Mitochondrial Homeostasis and Pink1/Parkin-Linked Mitophagy

Mitochondria are both major sources and targets of cellular oxidants. Their quality is maintained through coordinated control of biogenesis, dynamics, proteostasis, and selective mitochondrial autophagy, or mitophagy. Recent sesamin studies have focused predominantly on the PINK1/Parkin pathway.

In oleic acid-challenged HepG2 cells, sesamin reduced lipid accumulation, restored autophagy-associated activity, and improved indices of mitochondrial homeostasis [17]. Pharmacological autophagy inhibition attenuated the lipid-lowering effects of sesamin, providing functional support for the involvement of autophagy. Sesamin also increased PINK1/Parkin-associated mitophagy markers [17]. On the evidence reported, however, causal support is stronger for autophagy as a whole than for an indispensable role of PINK1/Parkin specifically, because changes in pathway proteins do not by themselves establish pathway dependence.

Complementary findings were reported in L02 cells exposed to 500 μM elaidic acid (9-*trans*-C18:1) [42]. Sesamin concentrations above 32 μM significantly reduced cell viability; therefore, 32 μM was selected for the subsequent mechanistic experiments. Under this condition, sesamin reduced ROS and MDA, increased reduced glutathione (GSH), and partially restored adenosine triphosphate (ATP) production and mitochondrial membrane potential [42]. Transmission electron microscopy showed changes compatible with improved mitochondrial morphology and increased autolysosome abundance. Sesamin also increased PINK1 and Parkin abundance, promoted the conversion of microtubule-associated protein 1 light chain 3 (LC3)-I to LC3-II, and reduced sequestosome-1 (p62) abundance [42].

The same study connected the autophagy-associated response to TFEB-regulated lysosomal function. Sesamin increased TFEB nuclear localisation and lysosome-associated membrane protein 1 (LAMP1) abundance, whereas eltrombopag or MHY1485 reversed its lipid-lowering effect [42]. These pharmacological perturbations support the involvement of TFEB-regulated autophagy–lysosome signalling. Nevertheless, because these compounds can affect processes beyond TFEB and the experiments were restricted to one in vitro lipid-stress model, they do not establish a linear sesamin–TFEB–PINK1/Parkin pathway or demonstrate efficacy against hepatic steatosis in vivo.

Together, the oleic acid and elaidic acid studies support an association between sesamin, mitochondrial preservation, and autophagy/mitophagy-related responses in lipid-loaded hepatic cell models [17,42]. The reported experiments do not directly establish the rate of mitochondrial cargo delivery to lysosomes using a pathway-specific mitophagy-flux assay. They also do not distinguish whether sesamin initially reduces mitochondrial oxidant production, modifies mitochondrial turnover, or produces both effects through interconnected feedback mechanisms.

### 3.3. Autophagic Flux and Lysosomal Control

The distinction between autophagosome abundance and autophagic flux is critical. An increase in LC3-II or autophagosome number can reflect either increased autophagosome formation or impaired lysosomal degradation. Conversely, a reduction in these markers can indicate suppression of autophagy initiation or more efficient completion of the pathway. Flux-based assays are therefore required before sesamin can be classified as an autophagy activator or inhibitor [43].

The strongest flux evidence among the available sesamin studies was provided by a 2025 investigation comparing sesamin, episesamin, and their monocatechol metabolites in human cell cultures expressing a tandem monomeric red fluorescent protein–GFP–LC3 (mRFP–GFP–LC3) reporter [14]. Both parent lignans and their metabolites increased autophagic flux, but the monocatechol metabolites produced stronger effects than their unmetabolised forms. Sesamin and its monocatechol metabolite inhibited mTORC1, decreased phosphorylation of ULK1 and TFEB, and promoted isolation-membrane formation and lysosomal biogenesis [14]. Mechanistic target of rapamycin complex 2 (mTORC2) activity was not detectably affected, supporting relative selectivity for mTORC1 under the conditions examined. This study provides stronger evidence for completed autophagic flux than experiments relying exclusively on LC3, p62, or autophagosome abundance, although it does not establish organ-specific flux or therapeutic effects in vivo.

The mTORC1–TFEB findings also provide a mechanistic connection between the 2025 metabolite study and the L02-cell study [14,42]. Inhibition of mTORC1 can simultaneously release ULK1-dependent autophagy initiation and TFEB-dependent lysosomal gene expression, thereby coordinating cargo sequestration with degradative capacity. The greater activity of the monocatechol metabolites further indicates that metabolism may qualitatively alter the effects attributed to administered sesamin.

Sesamin does not, however, regulate autophagy in a uniformly upward direction. Whereas the lipid-overload studies reported restoration or enhancement of autophagy-associated processes [14,17,42], sesamin attenuated the autophagy-associated response induced by diquat in HK-2 cells and mouse kidney [18]. This difference should not automatically be interpreted as a contradiction. Diquat-induced accumulation of autophagy markers may represent an excessive stress response or impaired downstream clearance, whereas lipid-loaded cells may exhibit insufficient organelle turnover. Without matched flux assays, lysosomal inhibition, and pathway-specific genetic rescue, the direction of marker changes cannot define whether autophagy is protective, maladaptive, or merely associated with the phenotype [43]. The current evidence is therefore more consistent with context-dependent modulation of autophagic homeostasis than with sesamin acting as a universal autophagy inducer.

### 3.4. Mitochondrial Danger Signalling and Integrated Interpretation

Damaged mitochondria can convert metabolic stress into innate immune signalling through mtDNA leakage. A 2026 study examined this mechanism in uterine smooth muscle cells (USMCs) exposed to palmitic acid and in pregnant mice fed a high-fat diet (HFD) [19]. High-fat exposure caused mitochondrial structural damage, ROS accumulation, and mtDNA leakage. These changes were accompanied by activation of cGAS and STING, increased interleukin-1β (IL-1β) and interleukin-18 (IL-18), and impaired insulin signalling [19]. Selective mtDNA depletion or STING knockdown attenuated these responses, providing functional evidence that mitochondrial damage was upstream of inflammatory and metabolic dysfunction.

Sesamin inhibited cGAS–STING activation, improved glucose uptake and insulin signalling, and enhanced mitochondrial respiratory function in the cellular model [19]. In pregnant HFD-fed mice, oral sesamin reduced systemic inflammation and improved uterine insulin sensitivity. Virtual screening and molecular docking predicted an interaction between sesamin and STING [19]. Because the proposed interaction was derived computationally, it should not be interpreted as demonstrating direct biochemical binding. Moreover, the findings were obtained in a specific pregnancy-associated metabolic model and have not been confirmed in human uterine tissue.

Collectively, the available evidence supports three interconnected levels of sesamin action. First, catechol metabolites can exhibit stronger radical-scavenging activity than the parent compound under cell-free conditions [12]. Second, sesamin may activate adaptive redox pathways, including NRF2 signalling in *Drosophila* and SIRT5/FOXO3a signalling in a mammalian toxic-injury model [18,40]. Third, sesamin and its metabolites can influence PINK1/Parkin-associated mitochondrial quality-control responses, mTORC1–TFEB-regulated autophagic flux, lysosomal biogenesis, and mtDNA–cGAS–STING signalling [14,17,18,19,42]. These processes may converge on a quality-control network in which reduced mitochondrial damage limits danger signalling, while autophagic and lysosomal pathways constrain the accumulation of dysfunctional organelles.

The strength of the evidence is nevertheless uneven. Tandem-reporter analysis provides comparatively strong support for autophagic flux [14], and mtDNA depletion and STING knockdown provide functional pathway validation in the uterine model [19]. By contrast, changes in PINK1, Parkin, LC3, p62, ROS-sensitive fluorescence, or antioxidant markers remain pathway-associated observations unless supported by flux measurements and pathway-specific rescue experiments. Among the studies considered here, no single experiment has simultaneously quantified parent sesamin and its metabolites, compartment-specific redox changes, mitochondrial respiration, mitophagic flux, lysosomal degradation, and functional rescue. The current evidence therefore supports sesamin as a conditional regulator of redox and organelle quality control rather than as a uniformly acting antioxidant. The principal experimental evidence linking sesamin to redox regulation and organelle quality control is summarised in Table 2.

## 4. Regulated Cell Death

Sesamin has been reported to suppress RCD in injured non-malignant tissues while promoting or sensitising RCD in tumour cells. This apparent bidirectionality is compatible with a context-dependent redox rheostat, but assignment of a specific death mechanism requires more than changes in viability or pathway-associated proteins. Current nomenclature distinguishes apoptosis, pyroptosis and ferroptosis according to their execution machinery and biochemical dependencies [5,6]. Accordingly, the evidence considered below is weighted most strongly when marker changes are accompanied by pharmacological rescue, pathway reactivation, gene silencing or tissue-specific gene deletion. Studies relying only on terminal markers are described as consistent with, rather than definitive for, a particular form of RCD.

### 4.1. Ferroptosis: Protection Across Toxic and Ischaemic Injury Models

Ferroptosis is driven by iron-dependent phospholipid peroxidation and failure of lipid-peroxide detoxification [5,6,24]. Robust identification therefore requires convergent evidence rather than reliance on individual pathway markers. Such evidence should ideally include demonstration of lipid peroxidation, iron dependence or altered labile iron, disruption of ferroptosis-relevant antioxidant systems, and functional rescue by ferroptosis-selective inhibitors or iron chelation. Genetic perturbation of key ferroptosis regulators and, where feasible, complementary morphological or lipidomic assessment can further strengthen mechanistic attribution. Altered GPX4, SLC7A11 or acyl-CoA synthetase long-chain family member 4 (ACSL4) alone is insufficient [24]. When evaluated against these criteria, recent sesamin studies provide several layers of preclinical support, although none establishes ferroptosis modulation in humans.

Two liver-injury studies implicated FOXO1. In perfluorooctanesulfonate-induced liver injury, sesamin reduced ferroptosis-related biochemical and molecular changes, whereas hepatocyte-specific *Foxo1* deletion abolished its protective effect on hepatocyte ferroptosis [21]. In a separate acetaminophen (APAP) study in male C57BL/6J mice (*n* = 6 per group), sesamin at 50 or 100 mg/kg/day was administered by gavage for 4 weeks before a single intraperitoneal APAP challenge at 300 mg/kg [44]. In AML12 and HepG2 cells exposed to 20 mM APAP, 40 μM sesamin and 2 μM ferrostatin-1 (Fer-1) each improved viability, whereas addition of 5 mM 3-methyladenine (3-MA) neutralised the protection conferred by sesamin. Hepatocyte-specific *Foxo1* deletion also eliminated sesamin-associated enhancement of autophagy and suppression of ferroptosis-related injury [44]. These interventions support a FOXO1-dependent link between autophagy competence and ferroptosis resistance under the tested toxicant conditions. They do not show that FOXO1 is a direct molecular target of sesamin or that the same sequence operates at nutritionally achievable human exposure.

Vascular and ischaemic models implicated different regulatory nodes. In apolipoprotein E-deficient mice, sesamin at 50 or 100 mg/kg/day for 12 weeks reduced atherosclerotic and endothelial-injury measures [22]. In human umbilical vein endothelial cells (HUVECs), pretreatment with 50 μM sesamin for 2 h before exposure to 100 μg/mL oxidised low-density lipoprotein for 24 h reduced ferrous iron, C11-BODIPY-detectable lipid oxidation and MDA, increased the reduced-to-oxidised glutathione ratio (GSH/GSSG), and shifted ACSL4, GPX4 and SLC7A11 in an anti-ferroptotic direction [22]. Fer-1 at 1 μM produced a similar protective phenotype. More importantly, *SREBF1* silencing weakened sesamin-mediated protection, whereas concurrent *METTL3* knockdown partially restored it, supporting an m^6^A-dependent METTL3–SREBF1 mechanism rather than a marker-only association [22].

In a rat random-pattern skin-flap study involving 54 animals, sesamin increased flap survival and was associated with activation of NRF2/SLC7A11/GPX4 signalling [23]. In HUVECs subjected to oxygen–glucose deprivation/reoxygenation, the NRF2 inhibitor ML385 weakened sesamin-induced protection, whereas Fer-1 served as a positive ferroptosis-control intervention [23]. This pharmacological evidence supports NRF2 dependence under the experimental conditions, although genetic confirmation and exposure measurements were not reported.

Renal APAP injury provides an additional, mechanistically distinct example. HK-2 cell experiments used 40 μM sesamin, 20 mM APAP, 50 μM hemin and 10 μM zinc protoporphyrin IX (ZnPP IX) [45]. Transcriptomic and biochemical analyses linked sesamin to restoration of *HMOX1* expression, which encodes HO-1, and attenuation of apoptosis- and ferroptosis-related changes. Hemin, used as an HO-1 inducer, reproduced key protective effects, whereas ZnPP IX substantially abolished sesamin-associated protection [45]. These findings support an HO-1-dependent protective component in this model, not a universal anti-ferroptotic role for HO-1, whose effects depend on cellular iron handling and redox context.

Collectively, the strongest ferroptosis evidence is provided by *Foxo1*, *SREBF1* and *METTL3* perturbation, together with Fer-1-sensitive phenotypes [21,22,44]. However, the studies span toxicant-exposed liver, APAP-exposed kidney, atherosclerotic endothelium and ischaemic skin, and implicate FOXO1, METTL3/SREBF1, NRF2 and HO-1, respectively [21,22,23,44,45]. These pathways should therefore be viewed as model-specific control points converging on lipid-peroxide restraint rather than as a single established sesamin receptor-to-ferroptosis pathway.

Importantly, the available evidence remains preclinical. Human studies of sesamin have not directly assessed ferroptosis-specific tissue endpoints integrating lipid peroxidation with iron dependence or ferroptosis-related antioxidant-system changes. Moreover, concentrations of parent sesamin and its metabolites have not been linked to ferroptosis-related changes in human target tissues, and no human study has established a causal relationship between sesamin exposure and ferroptotic cell death. These limitations currently preclude the conclusion that sesamin regulates ferroptosis in the human body.

### 4.2. Pyroptosis: Suppression in Metabolic Liver Injury but Promotion in Lymphoma

Pyroptosis is a lytic, pro-inflammatory RCD programme executed by gasdermin pores and commonly accompanied by inflammasome-dependent cytokine maturation [5,6]. In a mouse model of nonalcoholic steatohepatitis (NASH) induced by a high-fat, high-cholesterol diet (HFHCD; *n* = 10 per group), sesamin at 120 mg/kg/day by gavage for 6 weeks reduced hepatic NLRC4, cleaved caspase-1, the N-terminal fragment of gasdermin D (GSDMD-N) and mature IL-1β [20].

In primary mouse hepatocytes, exposure to 0.5 mM palmitate together with 50 or 100 μM sesamin for 48 h reduced lactate dehydrogenase release and the same pyroptosis-associated proteins [20]. In the palmitate plus 100 μM sesamin condition, phorbol 12-myristate 13-acetate (PMA; 200 nM) was applied for 12 h to reactivate PKC signalling. PMA increased PKCδ phosphorylation and attenuated sesamin-mediated suppression of the NLRC4–caspase-1–GSDMD axis [20]. This reversal experiment strengthens pathway attribution, but PMA is not selective for PKCδ, and the study did not use gasdermin- or inflammasome-deficient hepatocytes.

The direction was reversed in murine T-cell lymphoma. EL4 cells exposed to 10, 20 or 40 μM sesamin for 72 h showed autophagy before the appearance of apoptosis- and pyroptosis-associated changes, and sesamin also reduced tumour growth in mice [25]. Pharmacological inhibition of autophagy diminished both death phenotypes, supporting a contributory role for autophagy-associated processes [25]. Nevertheless, inhibition by an autophagy-modulating drug does not establish autophagy itself as the executing death mechanism, particularly without flux-selective genetic experiments [43]. The contrast between hepatocyte protection [20] and lymphoma-cell killing [25] is therefore better interpreted as context-dependent control of inflammasome and gasdermin signalling than as an intrinsic pro- or anti-pyroptotic property of sesamin.

### 4.3. Apoptosis-Related Responses: Cytoprotection in Organ Injury and Pro-Death Activity in Cancer

In non-malignant injury models, sesamin generally reduced apoptosis-associated damage. In diquat-induced acute kidney injury, its protection was linked to SIRT5/FOXO3a signalling and accompanied by suppression of oxidative stress, apoptosis and dysregulated autophagy [18]. In APAP nephrotoxicity, HO-1 inhibition substantially abolished sesamin-associated reductions in apoptosis- and ferroptosis-related injury [45]. These data support pathway-dependent cytoprotection, but neither study demonstrates that apoptosis suppression is independent of the broader reduction in toxicant burden and oxidative injury.

By contrast, the tumour models reviewed here generally reported apoptosis-associated responses or increased susceptibility to cell death, although usually at micromolar concentrations. In HeLa and SiHa cervical cancer cells, 75 or 150 μM sesamin for 24 h inhibited proliferation without materially affecting normal dermal fibroblasts at the same concentrations [26]. At 150 μM, the sub-G1 fraction reached 19.1 ± 2.4% in HeLa cells and 38.4 ± 2.7% in SiHa cells; annexin V-positive/propidium iodide-negative cells reached 21.3 ± 3.4% and 36.5 ± 4.5%, respectively [26]. Sesamin increased p53 phosphorylation and the abundance of p53 upregulated modulator of apoptosis (PUMA), BCL-2-associated X protein (BAX) and PTEN, while reducing protein kinase B (AKT) phosphorylation at Ser473. Pretreatment with 30 μM pifithrin-α for 2 h before exposure to 150 μM sesamin reduced apoptosis and restored proliferation, providing functional evidence for p53 involvement [26]. However, pharmacological p53 inhibition does not by itself prove direct activation of p53 by sesamin.

In A549 and CL1-5 lung adenocarcinoma cells, 100 μM sesamin combined with either 12.5 μM docetaxel or 5 μM paclitaxel reduced viability and colony formation more strongly than the individual treatments, with combination indices below 1 [27]. The combinations increased cleavage of caspase-3 and poly(ADP-ribose) polymerase (PARP), consistent with enhanced apoptosis [27]. Because no caspase-inhibitor rescue was reported for these combinations, the data demonstrate chemosensitisation accompanied by apoptotic signalling rather than exclusive apoptotic execution.

In nasopharyngeal carcinoma cells, 20 or 50 μM sesamin reduced proliferation and mitochondrial membrane potential, increased ROS and apoptosis-associated proteins, and altered autophagy markers [46]. Pretreatment with 5 mM NAC or 3 mM 3-MA for 2 h attenuated the effects of 50 μM sesamin during the subsequent 24 h exposure. In a xenograft experiment, 18 female BALB/c nude mice were divided equally among control, 50 mg/kg and 100 mg/kg sesamin groups and treated by daily gavage for 14 days. Sesamin reduced tumour volume and weight and increased cleaved caspase-3 [46]. These findings support contributions from oxidative signalling and autophagy, whereas the limited specificity of both inhibitors and the absence of a flux reporter preclude definitive causal ordering.

### 4.4. Integrated Interpretation

The available evidence supports a conditional rather than uniform effect of sesamin on RCD. Under toxic, metabolic or ischaemic stress, sesamin generally preserved non-malignant cells by limiting ferroptosis, pyroptosis or apoptosis [18,20,21,22,23,44,45]. In malignant cells, it promoted apoptosis, facilitated autophagy-associated apoptosis and pyroptosis, or increased chemotherapy sensitivity [25,26,27,46]. This pattern is consistent with differences in baseline redox state, mitochondrial reserve, tumour-suppressor competence and autophagic capacity, but no study has directly compared exposure-matched normal and malignant tissues within a single mechanistic design.

The evidential strength is uneven across these models. Tissue-specific or targeted genetic perturbation was used in the FOXO1 and METTL3/SREBF1 studies [21,22,44], whereas the NASH, skin-flap, cervical-cancer, APAP-nephrotoxicity and nasopharyngeal-carcinoma studies relied primarily on pharmacological interventions [20,23,26,45,46]. The diquat-kidney and lung-cancer studies were more dependent on associated signalling and terminal markers [18,27]. None of the RCD studies quantified intracellular parent sesamin or metabolite exposure at the site of pathway measurement [25,26,27,46]. Because sesamin metabolites may differ biologically from the parent compound [12,13,14,15], these findings cannot yet define clinically attainable cytoprotective or pro-death thresholds or assign between-model differences confidently to tissue identity, disease state, dose or metabolite exposure. The proposed intersections among redox regulation, organelle quality control and RCD are illustrated in Figure 2. The principal experimental evidence linking sesamin to regulated cell death is summarised in Table 3.

## 5. A Context-Dependent Redox-Rheostat Framework

Sesamin has been reported to exert cytoprotective effects in injured tissues while promoting cell death or chemosensitisation in selected tumour models. These apparently opposing effects cannot be explained adequately by classifying sesamin simply as an antioxidant or pro-oxidant. Biological redox regulation is spatially compartmentalised, kinetically controlled and dependent on the balance among oxidant production, antioxidant buffering, damage removal and stress-response capacity [1,2,3,4]. The term ‘redox rheostat’ is used here as a conceptual framework to describe how the net biological effects of sesamin may vary according to molecular exposure, metabolic identity, baseline cellular state, organelle quality control, and proximity to an RCD threshold (Figure 3).

This model does not imply that sesamin is itself a redox sensor, that it binds a single upstream rheostat protein or that a universal redox set point has been identified. It also should not be interpreted as evidence of hormesis. Demonstration of hormesis would require a reproducible biphasic response across a sufficiently broad concentration range within the same biological system. The current sesamin literature instead shows different response directions across heterogeneous cell types, disease models, concentrations and experimental durations. The rheostat concept consequently provides an organisational and hypothesis-generating framework rather than a quantitative dose–response law.

### 5.1. Exposure and Metabolic Identity Constitute the First Control Layer

The first determinant of the proposed rheostat is the identity and concentration of the molecular species reaching the relevant cellular compartment. Human pharmacokinetic data reported parent-sesamin and episesamin *C*_max_ values of approximately 7.6 and 54 nM after repeated administration of a 50 mg mixed-lignan preparation [15]. By contrast, mechanistic tumour studies principally used 10–150 μM parent sesamin, with one viability screen extending to 200 μM [25,26,27,46]. Relative to the reported parent-sesamin *C*_max_, these concentrations were approximately 1.3 × 10^3^ to 2.6 × 10^4^ times higher. This comparison demonstrates an exposure gap but does not equate total plasma concentration with the nominal concentration added to culture medium.

Chemical identity is equally important because catechol metabolites showed stronger radical-scavenging and ARE-activating activities and promoted greater autophagic flux than their parent lignans, while SC1 directly interacted with annexin A1 [12,13,14,41]. In PC12 cells, SC1 induced transient ROS–p38 MAPK–NRF2–HO-1 signalling, and NAC, dominant-negative NRF2 and zinc protoporphyrin attenuated components of this response [41]. These findings support metabolite-induced oxidative preconditioning followed by cytoprotection rather than simple ROS scavenging by parent sesamin.

Parent sesamin, episesamin and their metabolites therefore cannot be positioned at the same point on a single concentration axis or assumed to act through interchangeable mechanisms. A direct sequence from administered sesamin dose to target-tissue redox effect has not been established. The relevant input may be parent sesamin, a catechol metabolite, a conjugated species capable of cellular deconjugation, or a time-dependent mixture of these compounds. The active species may also differ among the liver, kidney, vascular endothelium, tumour tissue and intestinal environment. Although SC1 and EC1 are detectable in human plasma and several sesamin metabolites show greater activity than parent sesamin in experimental systems, current human data do not establish that these metabolites achieve higher pharmacologically active concentrations or preferential distribution in target tissues. Thus, their potentially greater biological relevance reflects functional nonequivalence rather than demonstrated superiority in human tissue exposure. Until parent compound and metabolite concentrations are measured in the same samples used for mechanistic analyses, pathway activation cannot be assigned confidently to orally administered sesamin itself.

### 5.2. Baseline Cellular State Determines the Direction of the Response

The second control layer is the redox and metabolic state of the responding cell before sesamin exposure. Across non-malignant injury models, sesamin-associated protection involved model-specific regulation of SIRT5/FOXO3a, cGAS–STING, PKCδ/NLRC4, FOXO1, METTL3/SREBF1, NRF2/SLC7A11/GPX4 and HO-1 [18,19,20,21,22,23,44,45]. These pathways do not constitute a demonstrated linear cascade; rather, they represent distinct routes through which damage generation, antioxidant defence or damage removal may shift cells away from an RCD threshold.

Malignant cells present a different but heterogeneous baseline. Many tumours maintain increased oxidant production while simultaneously enhancing antioxidant and metabolic systems that prevent ROS from reaching cytotoxic levels [47,48]. In this setting, additional oxidative pressure, impaired stress adaptation or interference with mitochondrial and autophagic capacity may move a tumour cell beyond its tolerable redox range. This interpretation is compatible with sesamin-associated ROS accumulation and apoptosis in nasopharyngeal carcinoma cells [46], p53/PTEN-dependent apoptosis in cervical cancer cells [26], chemotherapy sensitisation in lung adenocarcinoma cells [27], and autophagy-associated apoptosis and pyroptosis in murine lymphoma cells [25].

These cancer findings do not establish that sesamin acts as a direct chemical pro-oxidant. Increased ROS may arise secondarily from mitochondrial dysfunction, altered metabolic flux, autophagy perturbation or the execution of cell death itself. In the nasopharyngeal carcinoma study, attenuation of sesamin-associated effects by NAC supports the involvement of an oxidation-sensitive process but does not identify the ROS source or demonstrate direct ROS generation by sesamin [46]. Similarly, changes in p53, PTEN or AKT signalling identify responsive pathways but do not establish direct binding of sesamin to these proteins [26].

The distinction between non-malignant and malignant cells is therefore not absolute. HepG2 cells, although tumour-derived, displayed a protective PINK1/Parkin-associated mitophagy response when used as a model of oleic-acid-induced lipid accumulation [17]. PC12 cells are likewise tumour-derived, yet SC1 preconditioning protected them against H_2_O_2_-induced cell death through an NRF2/ARE- and HO-1-dependent response [41]. These findings indicate that the imposed stress, metabolic environment and measured outcome may be more influential than malignant origin alone. Conversely, activation of antioxidant programmes in a tumour could theoretically support survival or treatment resistance rather than promote death [47,48]. This possibility has not been tested adequately for sesamin and precludes assuming that low-dose antioxidant and high-dose anticancer effects represent a universal normal-cell–tumour-cell dichotomy.

### 5.3. Organelle Quality Control Forms a Major Inflection Point

Mitochondrial and autophagic competence may determine whether a sesamin-associated redox change is resolved adaptively or progresses towards RCD. In oleic-acid-treated HepG2 cells, sesamin-associated PINK1/Parkin-associated mitophagy response was linked to reduced lipid accumulation [17]. In APAP hepatotoxicity, inhibition of autophagy with 3-MA neutralised sesamin-associated protection, and hepatocyte-specific *Foxo1* deletion abolished both autophagy enhancement and suppression of ferroptosis-related injury [44]. These findings support a protective role for functional autophagic clearance under the specific injury conditions examined.

By contrast, autophagy contributed to tumour-cell death in the lymphoma and nasopharyngeal carcinoma models [25,46]. Pharmacological inhibition of autophagy attenuated sesamin-associated apoptosis and pyroptosis in EL4 cells [25] and weakened the effects of sesamin in nasopharyngeal carcinoma cells [46]. Monocatechol metabolites also enhanced autophagic flux more strongly than parent sesamin through selective mTORC1 inhibition [14]. Collectively, these observations position autophagy as a potential inflection point rather than as an intrinsically protective or cytotoxic process.

Because autophagy-associated protein abundance, autophagosome number and 3-MA-sensitive phenotypes do not independently establish increased flux or causal autophagy dependency, these interpretations require time-resolved flux assays and genetic perturbation at multiple autophagy stages [43].

Mitochondrial quality control and ferroptosis resistance may also be coupled rather than independent. Removal of damaged mitochondria can decrease oxidant generation, whereas preservation of GSH-dependent lipid-peroxide detoxification limits membrane damage and ferroptotic execution. Nevertheless, the relative contribution of mitochondrial ROS to ferroptosis remains model-dependent [24], and the available sesamin studies did not simultaneously quantify mitochondrial redox state, phospholipid oxidation, autophagic flux and death rescue. The proposed connection between mitophagy and ferroptosis resistance should therefore remain conditional rather than be presented as a universal sequence.

### 5.4. Upper and Lower Boundaries of the Rheostat

The proposed model requires both an oxidative and a reductive boundary. At the oxidative boundary, sustained oxidant generation, lipid peroxidation, mitochondrial failure or inflammatory amplification exceeds cellular repair and buffering capacity, promoting apoptosis, pyroptosis or ferroptosis. Sesamin-associated tissue protection would move an injured cell away from this boundary by reducing damaging inputs, supporting antioxidant defence or improving organelle quality control [18,20,21,22,23,44,45]. Conversely, the high nominal sesamin concentrations used in tumour experiments may increase cellular stress or weaken adaptive capacity sufficiently to move selected cancer cells towards an RCD threshold [25,26,27,46].

At the opposite boundary, excessive or persistent reducing capacity can disrupt physiological redox signalling, metabolic flexibility and protein homeostasis. Reductive stress has also been implicated in tumour adaptation and survival [49]. No cited sesamin study, however, demonstrated reductive stress through coordinated measurement of redox couples, reducing equivalents and redox-sensitive protein modifications. Increased GSH, antioxidant-enzyme expression or NRF2-associated signalling alone should therefore not be interpreted as evidence that sesamin induces reductive stress.

NRF2 is also context-dependent: its activation can preserve stressed cells, whereas persistent activity may support tumour survival and treatment resistance [47,48]. Functional experiments support NRF2 involvement in SC1-treated PC12 cells [41] and sesamin-treated ischaemic endothelial models [23]. However, these studies examined different molecular species and experimental systems and do not establish a universal NRF2 mechanism after oral sesamin administration in humans.

### 5.5. Testable Predictions and Requirements for Validation

For experimental purposes, the proposed rheostat can be operationalised using four measurable components: (i) molecular exposure, defined by intracellular concentrations of parent sesamin and relevant metabolites; (ii) baseline redox state, assessed using complementary measures of oxidant burden and reducing capacity; (iii) organelle adaptive reserve, reflected by mitochondrial function, mitophagic or autophagic flux, and lysosomal competence; (iv) proximity to regulated cell-death commitment or execution, assessed using pathway-specific biochemical and rescue criteria. Current evidence is insufficient to define universal numerical cut-offs for these variables. Quantitative validation should therefore focus on whether controlled alteration of one component reproducibly shifts the sesamin concentration or exposure duration required to produce cytoprotection or regulated cell death within the same biological system.

The rheostat model generates several experimentally testable predictions. First, exposure-matched normal and malignant cells should respond differently only when their baseline redox state, mitochondrial reserve or adaptive capacity differs. Second, parent sesamin and its major metabolites should produce distinguishable pathway and RCD profiles when tested at matched intracellular concentrations. Third, genetic disruption of a proposed control node should shift the concentration or duration required for sesamin to produce protection or death rather than merely alter a terminal marker. Fourth, restoration of the disrupted pathway should reverse the effect of the genetic perturbation. Fifth, concentrations approximating human parent-compound exposure should not be assumed to reproduce effects observed at high micromolar concentrations unless intracellular accumulation or active-metabolite exposure is demonstrated.

Testing these predictions requires exposure-matched systems that quantify parent sesamin and its metabolites, resolve redox and organelle responses over time, and use pathway-specific rescue and genetic perturbation [3,5,6,24,43]. Matched primary cells, organoids or co-culture systems should compare normal and diseased tissues at identical extracellular and measured intracellular exposures. Only a reproducible switch between protection and death within a harmonised design would permit quantitative definition of a sesamin-associated redox rheostat.

The redox-rheostat model integrates the current literature without assigning sesamin an invariant antioxidant or pro-oxidant identity. It remains an unvalidated organising framework whose principal value is to distinguish genuine context dependence from differences in exposure, model selection and assay design.

## 6. Translational Relevance and Priorities for Clinical Development

The human evidence for sesamin remains limited to small, short-duration intervention studies that assessed pharmacokinetics, tolerability, cardiovascular or metabolic risk factors, inflammatory biomarkers and subjective sleep outcomes. None of the human studies identified for this review directly measured concentrations in target tissues, demonstrated pathway dependency or evaluated an RCD mechanism. The available findings should therefore be interpreted as preliminary evidence concerning exposure and selected surrogate outcomes rather than proof of disease modification or clinical efficacy.

### 6.1. Human Exposure, Tolerability and Intervention Studies

The principal repeated-dose study provides the main short-term safety evidence: 24 healthy participants received a 50 mg/day sesamin/episesamin preparation for 28 days, with no parent-lignan accumulation or serious adverse events reported [15]. These findings apply only to this mixed-lignan preparation and do not establish the safety of isolated sesamin, higher doses, prolonged administration, or use in patients with hepatic or renal impairment.

A double-blind, placebo-controlled crossover study included 25 middle-aged participants with mild hypertension [50]. The active preparation provided 60 mg/day of a mixture containing sesamin and episesamin at an approximately 1:1 molar ratio. Each intervention period lasted 4 weeks and was separated by a 4-week washout period [50]. During the active period, systolic blood pressure decreased from 137.6 ± 2.2 to 134.1 ± 1.7 mmHg (*p* = 0.044), whereas diastolic blood pressure decreased from 87.7 ± 1.3 to 85.8 ± 1.0 mmHg (*p* = 0.045). The corresponding values during the placebo period were 135.0 ± 1.8 to 135.1 ± 1.7 mmHg (*p* = 0.92) and 85.9 ± 1.2 to 86.6 ± 1.2 mmHg (*p* = 0.48), respectively [50]. These results represent pre-intervention to post-intervention comparisons within each period rather than a reported treatment-by-period effect estimate. The small sample and short intervention period further limit conclusions about antihypertensive efficacy.

In a randomised, double-blind, placebo-controlled trial, 48 participants with type 2 diabetes received either sesamin at 200 mg/day or placebo for 8 weeks [51]. Relative to placebo, sesamin reduced fasting blood glucose, haemoglobin A1c, and tumour necrosis factor-α (TNF-α), with all comparisons reported as *p* < 0.05 [51]. Additional decreases in waist circumference, hip circumference, body adiposity index, TNF-α, and interleukin-6 (IL-6), together with an increase in adiponectin, were reported as within-group changes. The short duration, small sample, and absence of clinical complication outcomes limit conclusions concerning durability and clinical efficacy.

Two publications arose from the same registered 6-week trial involving 44 women with rheumatoid arthritis who received sesamin at 200 mg/day or placebo [52,53]. In the cardiovascular-risk analysis, significant between-group differences were reported for MDA (*p* = 0.018), total antioxidant capacity (*p* = 0.020) and high-density lipoprotein cholesterol (HDL-C; *p* = 0.007) [52]. Weight, body mass index, waist-to-hip ratio, body fat, systolic blood pressure, triglycerides, total cholesterol (TC) and low-density lipoprotein cholesterol (LDL-C) changed significantly from baseline in the sesamin group, but the corresponding between-group differences were not significant [52]. A subsequent publication from the same trial reported lower high-sensitivity C-reactive protein and TNF-α relative to placebo. Hyaluronidase and matrix metalloproteinase-3 decreased in the sesamin group, and reductions in tender-joint count and pain severity were also reported [53]. Because both publications analysed the same participant cohort, they should not be treated as two independent clinical trials.

A double-blind, self-controlled crossover study investigated sesamin at 94.0 mg/day in participants with low-arousal-threshold obstructive sleep apnoea or a risk of obstructive sleep apnoea [54]. Thirty-three participants were enrolled, 25 completed the study and 20 were included in the statistical analysis. Each intervention period lasted 8 weeks and was separated by a 2-week washout period [54]. During sesamin administration, Pittsburgh Sleep Quality Index scores decreased from 7.4 ± 3.4 to 6.4 ± 3.2 and Epworth Sleepiness Scale scores decreased from 5.6 ± 3.9 to 3.7 ± 3.8. However, the changes did not differ significantly from those during the placebo period, with *p* = 0.6433 and *p* = 0.4063, respectively [54].

Plasma glutathione peroxidase (GPX) activity increased from 162.0 ± 39.4 to 194.9 ± 67.2 nmol/min/mL during sesamin administration [54]. The mean change was 32.97 ± 55.99 nmol/min/mL during the sesamin period and −7.83 ± 65.85 nmol/min/mL during the placebo period, producing a significant between-period difference (*p* = 0.0215). Plasma MDA and erythrocytic GSH and glutathione reductase changed significantly within the sesamin period, but their between-period comparisons were not significant, with *p* values of 0.1327, 0.1358 and 0.5547, respectively [54]. This study therefore identified an isolated placebo-controlled change in plasma GPX activity but did not demonstrate a placebo-controlled improvement in subjective sleep outcomes or a consistent effect across multiple redox biomarkers.

### 6.2. Interpretation of Pooled Clinical Evidence

A systematic review and meta-analysis included seven randomised trials with a combined total of 212 participants [55]. Sesamin-related interventions were associated with reductions in TC (weighted mean difference [WMD], −10.893 mg/dL; 95% confidence interval [CI], −19.745 to −2.041; *p* = 0.016; *I*^2^ = 49.8%) and LDL-C (WMD, −8.429 mg/dL; 95% CI, −16.086 to −0.771; *p* = 0.031; *I*^2^ = 53.3%). Systolic blood pressure was also reduced (WMD, −3.662 mmHg; 95% CI, −6.220 to −1.105; *p* = 0.005; *I*^2^ = 20.8%) [55]. No significant pooled effects were detected for HDL-C, triglycerides, diastolic blood pressure or body weight.

These pooled estimates require cautious interpretation. The included studies comprised only 212 participants, intervention durations ranged from 28 to 60 days and individual outcomes were generally contributed by fewer than seven trials [55]. The preparations were also heterogeneous and included isolated or mixed sesame lignans, sesame-containing interventions and at least one intervention combining sesame lignans with vitamin E. Consequently, the pooled estimates cannot be interpreted as effects attributable exclusively to chemically defined parent sesamin. The modest effect sizes, limited number of studies and moderate heterogeneity for TC and LDL-C further preclude conclusions about long-term cardiovascular benefit. The principal human intervention studies and pooled clinical evidence are summarised in Table 4.

### 6.3. Safety and Interaction Boundaries

The available intervention studies provide only short-term safety information. The most detailed repeated-dose study evaluated 50 mg/day of a sesamin and episesamin mixture for 28 days in healthy adults [15], whereas studies using 200 mg/day in participants with type 2 diabetes or rheumatoid arthritis lasted only 6–8 weeks [51,52,53]. These trials were not sufficiently large or prolonged to identify uncommon adverse events, cumulative toxicity or risks in vulnerable populations. Safety has not been established during pregnancy, lactation, childhood, advanced age with multimorbidity, hepatic or renal impairment or concurrent treatment with multiple medicines.

The potential for food–drug interactions also remains unresolved. Sesamin inhibited CYP2C9 and CYP2C19 in recombinant human enzyme systems, and model-based calculations identified phenytoin as a potentially relevant narrow-therapeutic-index substrate [39]. However, these findings are based on in vitro inhibition and risk prediction rather than a pharmacokinetic interaction demonstrated in humans. Dietary sesame intake, concentrated sesamin supplements and exposure-enhancing formulations should not be assumed to carry equivalent interaction risks. Controlled human interaction studies are required before clinically actionable recommendations can be made.

### 6.4. Priorities for Clinical Development

Future trials should use chemically characterised interventions and report the quantities of sesamin, episesamin, other sesame lignans, excipients and formulation components. Dose selection should be supported by human pharmacokinetic data rather than by direct conversion from animal doses or by nominal concentrations used in cell culture. Studies should measure parent sesamin together with major unconjugated and conjugated metabolites, incorporate serial sampling and report unbound concentrations when technically feasible [15].

Mechanistic clinical studies should distinguish circulating biomarker changes from tissue target engagement and prespecify the evidence required for pathway attribution [3,4,24,43]. Changes in NRF2-related proteins, GPX activity, MDA or inflammatory mediators alone should not be interpreted as causal mechanisms.

Clinical studies should be adequately powered, randomised, preregistered and designed with prospectively specified primary outcomes [56]. Parallel dose groups and sufficiently long follow-up are needed to define dose–exposure–response relationships, persistence of effects and delayed adverse events. Relevant sources of heterogeneity include age, sex, baseline metabolic or inflammatory status, habitual sesame intake, microbiota-dependent metabolism, CYP genotype, concomitant medication and formulation-dependent bioavailability. Cancer studies require particular caution because no human trial has demonstrated antitumour efficacy, chemosensitisation or selective induction of tumour-cell death by sesamin. Clinical development should therefore proceed from exposure-confirmed mechanistic studies to disease-specific efficacy trials rather than extrapolating directly from high-concentration cell-culture experiments.

## 7. Methodological Limitations and Future Research Directions

### 7.1. Chemical Identity and Exposure Relevance

Oral administration does not expose tissues to a single pharmacologically stable entity but to a changing mixture of parent sesamin, episesamin, catechol metabolites, conjugated derivatives and microbiota-derived enterolignans with distinct pharmacokinetic and biological properties [12,13,14,15,28,29,30,31,32,33,34,35]. Experiments reporting only nominal parent-sesamin concentrations cannot identify the active species when the model retains metabolic or deconjugating capacity.

Future studies should specify compound purity, stereochemical composition, formulation, vehicle, storage conditions and stability in the experimental medium. Parent sesamin, episesamin and their major metabolites should be examined separately and in exposure-defined combinations. Validated mass-spectrometric methods should quantify extracellular, intracellular and, where possible, subcellular concentrations over time. Protein binding and cellular uptake should also be considered because the nominal concentration added to culture medium may differ substantially from the unbound concentration available to the cell.

Experimental concentrations should be justified against measured human plasma or tissue exposure [15,26,27]. High-concentration experiments may help identify stress responses, but claims of pharmacological relevance require direct exposure data. Concentration, exposure duration and concentration–time profile should be reported separately because a short high-concentration pulse is not equivalent to prolonged low-concentration exposure.

Species differences further complicate translation. The relative contributions of catecholisation and glucuronidation differ between rat and human liver preparations, and sesamin and episesamin undergo distinct CYP-dependent metabolic pathways [31,32,33]. Conjugated metabolites may also accumulate in tissues or undergo cell-specific deconjugation [34,35]. Animal studies should therefore measure the molecular species present in plasma and target tissues rather than infer exposure from the administered dose. Comparison of effective tissue exposure with human pharmacokinetic data is essential before proposing clinical relevance.

### 7.2. Redox Measurements and Pathway Attribution

Many sesamin studies infer an antioxidant or pro-oxidant effect from a small number of global measurements. General oxidation-sensitive fluorescent probes, MDA, total antioxidant capacity and changes in antioxidant-enzyme expression can provide supporting information, but none independently identifies the oxidant species, its source, its subcellular location or the direction of redox signalling [1,2,3,4]. A decrease in probe fluorescence, for example, may reflect altered probe loading, metabolism, efflux or cell number rather than a direct reduction in ROS.

Redox experiments should combine orthogonal measurements of oxidant generation, antioxidant capacity and molecular damage. Whenever feasible, studies should identify the relevant oxidant species and distinguish mitochondrial, cytosolic, lysosomal and membrane-associated redox changes. Genetically encoded compartment-targeted sensors, direct measurements of oxidative modifications and assessment of oxidant-producing enzymes can provide greater spatial and mechanistic resolution [57]. Results should be normalised to viable cell number or another appropriate measure when sesamin alters proliferation or survival.

Temporal resolution is also important. An early, transient increase in ROS may initiate adaptive signalling without producing sustained oxidative damage, whereas prolonged oxidant accumulation may contribute to RCD [58]. Single end-point measurements cannot distinguish these possibilities. Time-course experiments should relate changes in ROS, redox-sensitive signalling, antioxidant responses, organelle dysfunction and cell death within the same model.

NRF2 activation should not be inferred solely from increased total NRF2 protein or increased *HMOX1* expression. Stronger evidence requires assessment of NRF2 stabilisation and nuclear localisation, ARE-dependent transcription, multiple target genes and loss of the response following genetic or pharmacological disruption of the pathway [59]. Rescue or re-expression experiments are needed when NRF2 dependency is claimed. This distinction is particularly important because SC1 and EC1 were stronger ARE activators than their parent compounds, whereas the functional experiments focused primarily on SC1 [41]. Findings obtained with SC1 should therefore not be assigned automatically to parent sesamin, episesamin or other metabolites.

### 7.3. Organelle Quality Control and Autophagy

Changes in mitochondrial membrane potential, ATP content, morphology or selected mitochondrial proteins do not individually demonstrate improved mitochondrial quality control. Studies should combine measurements of respiration, membrane potential, ATP production, mitochondrial ROS, morphology and turnover. When mitophagy is proposed, mitochondrial delivery to lysosomes and completion of degradation should be demonstrated rather than inferred from changes in PINK1, Parkin or LC3 abundance alone.

Autophagy requires particularly careful interpretation. An increase in LC3-II or autophagosome number may indicate enhanced autophagosome formation, impaired lysosomal degradation or both. Conversely, a reduction in p62 abundance cannot establish increased flux when its transcription or synthesis is altered. Autophagic flux should be assessed using lysosomal inhibition, tandem fluorescent reporters or equivalent approaches that distinguish autophagosome formation from degradation [43,60]. Genetic disruption of essential autophagy components should then determine whether autophagy is required for the observed cytoprotective or cytotoxic effect.

The recent finding that sesamin and episesamin monocatechol metabolites inhibit mTORC1 and enhance autophagic flux more strongly than the parent lignans illustrates why metabolite identity and flux measurements must be integrated [14]. Future work should determine whether the metabolite concentrations required for this response are achieved in relevant tissues and whether mTORC1 inhibition is necessary and sufficient for the downstream phenotype. Autophagy should not be labelled protective or detrimental solely from its association with improved survival or increased cell death because it may represent an adaptive response, a permissive process or an accompanying marker rather than the direct cause of the outcome.

### 7.4. Establishing the Identity and Causality of Rcd

The classification of RCD should follow mechanism-based criteria rather than reliance on a limited set of proteins or biochemical markers [5,6]. Apoptosis claims should integrate morphological evidence, caspase activation, substrate cleavage and rescue through relevant genetic or pharmacological interventions. Changes in BAX, B-cell lymphoma 2 protein (BCL-2) or cleaved caspase abundance alone do not demonstrate that apoptosis is the dominant mechanism responsible for loss of viability.

Pyroptosis should be supported by activation of the relevant inflammatory caspase, cleavage of the responsible gasdermin, membrane permeabilisation and lytic cell death. Inflammasome-protein expression or cytokine release alone is insufficient because these responses can occur without pyroptotic execution. Genetic depletion or inhibition of the proposed gasdermin or upstream caspase should attenuate the death phenotype before causal dependency is concluded.

Ferroptosis requires evidence of iron-dependent phospholipid peroxidation and suppression of cell death by mechanistically appropriate ferroptosis inhibitors or iron chelation [24]. Increased MDA, altered iron content, decreased GPX4 expression or changes in SLC7A11 do not individually establish ferroptosis. Studies should incorporate lipid-peroxidation measurements, rescue experiments and genetic perturbation of the proposed regulatory pathway. Changes in ferroptosis-associated proteins should be distinguished from demonstration that ferroptosis accounts for the observed tissue injury or loss of cell viability.

The coexistence of several death-related signals should not automatically be interpreted as simultaneous execution of multiple RCD programmes. Stress responses, pathway cross-talk and secondary activation after membrane disruption can produce overlapping molecular signatures [61]. Time-resolved experiments and independent rescue of each pathway are needed to determine whether apoptosis, pyroptosis, ferroptosis or another process is dominant, sequential or dispensable. Because commonly used inhibitors can have off-target effects, conclusions should be supported by more than one pharmacological agent and, where feasible, by genetic validation.

### 7.5. Biological Context, Model Selection and Reproducibility

The proposed redox-rheostat model predicts that sesamin responses depend on baseline cellular state rather than disease label alone. This hypothesis should be tested directly by measuring the variables expected to influence the response, including basal ROS, antioxidant capacity, mitochondrial function, labile iron, lipid composition, autophagic competence and proximity to an RCD threshold. Comparisons should include matched normal and tumour cells, treatment-sensitive and treatment-resistant cells, and models with defined differences in redox or metabolic capacity. The effect of sesamin should then be related quantitatively to baseline state rather than interpreted only from comparisons between unrelated cell lines.

Culture conditions can substantially modify redox metabolism and treatment response. Oxygen tension, glucose concentration, serum composition, antioxidant content, cell density and exposure duration should therefore be standardised and reported. Cell lines should be authenticated and tested for contamination, and findings should be reproduced in independent models. Primary cells, patient-derived organoids and multicellular systems may provide greater physiological relevance, particularly when metabolism, immune signalling or tissue-specific deconjugation is central to the proposed mechanism.

Animal studies should incorporate randomisation, allocation concealment, blinded outcome assessment, predefined exclusion criteria and sample-size justification. Both sexes should be considered unless a clear biological reason supports restriction to one sex. Dose selection should be linked to measured plasma and tissue exposure, and pharmacodynamic measurements should be obtained at time points aligned with the presence of parent sesamin or its metabolites. Reporting only the administered dose and a terminal tissue phenotype is insufficient to establish an exposure–mechanism–outcome relationship. The validity of the disease model itself should also be considered when interpreting sesamin-associated mechanisms. Toxicant-induced liver or kidney injury, diet-induced metabolic disease, ischaemic tissue models, xenografts, and other experimental systems reproduce only selected aspects of the corresponding human conditions and may differ in disease duration, immune context, metabolic state, and baseline redox environment [62]. Consequently, pathway involvement demonstrated within a specific animal model should not be assumed to represent a universal mechanism in human disease. Mechanistic conclusions should therefore be interpreted as model-dependent unless they are reproduced across complementary systems and supported by exposure data relevant to humans.

### 7.6. An Integrated Experimental Roadmap

A rigorous research sequence should begin by identifying the sesamin-related molecular species present in the relevant biological compartment. The second step should determine whether those species reproduce the proposed response at experimentally and clinically relevant concentrations. Time-resolved and compartment-specific measurements should then establish the order of redox changes, organelle responses and RCD. Genetic perturbation, orthogonal pharmacological intervention and rescue experiments should determine causal dependency. Finally, the mechanism should be confirmed in an exposure-verified animal or human-derived model before progression to clinical testing.

The redox-rheostat model should be considered supported only if baseline state predicts response direction across independent systems and deliberate modification of that state changes the sesamin response. Until then, it remains an organising hypothesis rather than a validated predictive mechanism.

## 8. Conclusions

Sesamin cannot be understood adequately as a conventional antioxidant with a uniform biological effect. Oral exposure generates parent sesamin, episesamin, catechol metabolites, conjugated derivatives, and microbiota-derived products that differ in pharmacokinetics and biological activity. Available evidence indicates that these molecular species can influence redox signalling, mitochondrial quality control, autophagy, and regulated cell death. In non-malignant injury models, sesamin-related responses are generally associated with cytoprotection, whereas in selected tumour models sesamin may increase oxidative stress, disrupt survival signalling, or enhance susceptibility to cell death. These divergent outcomes are consistent with a context-dependent redox-rheostat framework influenced by molecular exposure, metabolic competence, baseline redox state, organelle reserve, and proximity to a regulated cell-death threshold. However, this framework remains hypothesis-generating rather than a validated predictive mechanism.

Translation to humans remains limited by sparse tissue-exposure data, frequent use of pharmacologically unverified micromolar concentrations, and incomplete pathway validation. Future studies should therefore integrate quantitative measurement of parent compounds and metabolites with exposure-relevant experimental designs, organelle-flux assays, and pathway-specific rescue or genetic approaches. Such studies will be necessary to determine whether sesamin acts primarily as a dietary lignan with modest biological effects, a precursor of active metabolites, or a clinically relevant modulator of redox regulation and cell fate.

## Figures and Tables

**Table 1:** Key pharmacokinetic, metabolic, and interaction evidence relevant to sesamin exposure and biological interpretation.

Evidence/Model	Exposure/System	Key Findings	Interpretation/Limitation	Ref.
Human repeated-dose pharmacokinetics	Healthy adults; 50 mg/day sesamin/episesamin (1:1) for 28 days; *n* = 48, active *n* = 24, PK subset *n* = 10	Sesamin, episesamin, SC1, and EC1 reached *T*_max_ at ~5 h. Parent-sesamin and episesamin *C*_max_ were ~7.6 and 54 nM. SC1, EC1, and episesamin *t*_1/2_ were 2.4, 3.4, and 7.1 h, respectively. No parent-lignan accumulation was detected after day 7.	Principal human exposure study, but based on a mixed-lignan preparation, small PK subset, and no tissue measurements.	[15]
Human urinary elimination	Six healthy participants consumed sesame oil containing 508 μmol (~180 mg) sesamin	Urinary recovery of a sesamin monocatechol metabolite was 22.2–38.6% of intake (mean 29.3 ± 5.6%), mainly within 12 h.	Enzymatic hydrolysis before analysis prevented distinction between free and conjugated metabolites.	[28]
Human and microbial enterolignan formation	Four participants consumed sesame seeds; sesamin was also incubated with human faecal microbiota	Sesamin was converted to enterolactone; faecal incubation identified enterolactone as the principal end product and three intermediates.	Very small human sample and marked interindividual variability limit generalisation.	[29]
Human–cell culture exposure comparison	Human parent-sesamin *C*_max_ ~7.6 nM versus 75–150 μM in cervical cancer cells and up to 100 μM in lung adenocarcinoma cells	Cancer-cell concentrations were several orders of magnitude above reported human parent-sesamin plasma exposure.	Nominal culture concentrations do not equal unbound intracellular or tissue exposure; comparable human tissue levels have not been demonstrated.	[15,26,27]
Initial hepatic monocatechol formation	Recombinant human CYP enzymes and human liver microsomes	Predicted hepatic contribution: CYP2C9 > CYP1A2 > CYP2C19 > CYP2D6; CYP2C9 was the principal contributor in microsomal analyses.	Derived from in vitro systems; relative contributions have not been quantified in vivo in humans.	[31]
Downstream hepatic and stereochemical metabolism	Human/rat liver microsomes, recombinant human UGTs, and human liver cytosol	Human microsomes favoured glucuronidation over further catecholisation; UGT2B7 predominated in glucuronidation. COMT-dependent methylation occurred. Episesamin metabolism differed stereochemically from sesamin.	Demonstrates species- and stereochemistry-dependent metabolism; sesamin and episesamin are not pharmacokinetically interchangeable.	[32,33]
Rat mass balance and tissue distribution	Single oral 5 mg/kg [^14^C]sesamin	Plasma radioactivity peaked at 1 h; *t*_1/2_ 4.7 h. Recovery: urine 37.5 ± 3.1%, faeces 58.7 ± 4.8%; bile-duct-cannulated rats: bile 66.3 ± 8.4%, urine 27.8 ± 10.2%. Liver and kidney showed prominent exposure, mainly as conjugates.	Measures total sesamin-derived radioactivity, not unchanged sesamin; rat distribution cannot be directly extrapolated to humans.	[30]
Sulfate-conjugate formation	Rats given 100 mg/kg sesamin; rat/human liver cytosol and recombinant human SULTs	Two SC1 sulfate conjugates showed *C*_max_ values ~5- and 10-fold higher than parent sesamin in rats. Sulfation efficiency was 1.7-fold higher in human than rat liver cytosol.	Supports human sulfate-forming capacity, but individual sulfate conjugates have not been quantified in vivo in humans.	[34]
Cellular deconjugation and intracellular metabolism	J774.1 macrophage-like cells treated with SC1 glucuronides	Effects were associated with intracellular SC1 accumulation; β-glucuronidase inhibition abolished and COMT inhibition enhanced the response. IFN-β and iNOS were suppressed.	Supports cell-specific deconjugation but not universal glucuronide-mediated delivery in human tissues.	[35]
Parent compound–metabolite functional nonequivalence	Cell-free, cellular, and animal systems	Catechol metabolites showed stronger radical-scavenging activity; SC1 showed annexin A1-dependent anti-inflammatory activity; monocatechol metabolites enhanced autophagic flux more strongly through mTORC1 inhibition.	Biological activity depends on molecular species and experimental context; human tissue exposure remains uncertain.	[12,13,14]
Formulation-dependent oral exposure	Rats; slowly self-emulsifying sesamin formulation, 20 mg/kg	AUC was 7.9-fold greater than crystalline sesamin; *C*_max_ was 22% lower and mean residence time increased to 5.3 h versus a conventional self-emulsifying formulation.	Demonstrates formulation-dependent exposure in rats; human efficacy and pharmacokinetic benefit remain unestablished.	[36]
CYP2C9 inhibition and diclofenac pharmacokinetics	Human liver microsomes; rats pretreated with 10 or 100 mg/kg sesamin	Sesamin produced mechanism-based CYP2C9 inhibition in vitro, but the intermediate complex was unstable. Diclofenac *C*_max_, *T*_max_, AUC, and hepatic hydroxylation were unchanged in rats.	Negative rat findings do not exclude a human interaction.	[31,37]
*S*-Warfarin 7-hydroxylation inhibition	Human liver microsomes and recombinant CYP2C9	Reported *K*_i_ values differed markedly: 202 ± 18 nM versus 15.7 μM in human liver microsomes; recombinant CYP2C9 *K*_i_ was 13.1 μM.	~78-fold discrepancy between microsomal estimates prevents reliable clinical extrapolation; no human PK interaction was tested.	[16,38]
Recombinant enzyme interaction screen	Sesamin 0, 1, 10, and 100 μM with recombinant human drug-metabolising enzymes	No significant inhibition of examined UGTs; CYP2C9 and CYP2C19 were inhibited. Model-based assessment highlighted phenytoin.	Demonstrates in vitro interaction potential only; clinical relevance remains unconfirmed.	[39]

Abbreviations: AUC, area under the concentration–time curve; COMT, catechol-*O*-methyltransferase; *C*_max_, maximum plasma concentration; CYP, cytochrome P450; EC1, episesamin-derived monocatechol metabolite; IFN-β, interferon-β; iNOS, inducible nitric oxide synthase; *K*_i_, inhibition constant; mTORC1, mechanistic target of rapamycin complex 1; PK, pharmacokinetic; SC1, sesamin-derived monocatechol metabolite; SULT, sulfotransferase; *T*_max_, time to maximum plasma concentration; *t*_1/2_, terminal half-life; UGT, uridine diphosphate-glucuronosyltransferase.

**Table 2:** Experimental evidence linking sesamin to redox regulation and organelle quality control.

Mechanistic Level	Model/Stressor	Sesamin Intervention	Key Findings	Validation/Limitation	Ref.
Direct redox chemistry	Cell-free enzymatic metabolic systems	Parent sesamin and enzymatically generated mono- and dicatechol metabolites	Parent sesamin showed no detectable antioxidative activity, whereas catechol metabolites showed strong radical-scavenging activity.	Supports metabolite-dependent redox activity but does not establish that the same reactions predominate in intact cells or human tissues.	[12]
Adaptive redox signalling	*Drosophila* larvae carrying an ARE–GFP reporter	Dietary parent sesamin	Sesamin activated Cap ‘n’ collar isoform C in the CNS, gut, and salivary glands and increased *Cyp6a2* and *Cyp6g1* transcripts.	Glial *Keap1* expression attenuated reporter activation, supporting NRF2-dependent signalling; mammalian target engagement was not established.	[40]
Metabolite-dependent adaptive redox signalling	PC12 cells exposed to H_2_O_2_	SC1 and EC1 versus parent lignans	SC1 and EC1 activated ARE signalling more strongly than parent compounds; SC1 increased NRF2 nuclear translocation and HO-1 expression.	N-acetyl-L-cysteine blocked SC1-induced p38 MAPK phosphorylation and HO-1 induction; zinc protoporphyrin and dominant-negative NRF2 reduced cytoprotection. Evidence is limited to a rat cell line.	[41]
Toxicant-induced redox and mitochondrial injury	Diquat-exposed HK-2 cells and mice with diquat-induced AKI	Parent sesamin	Sesamin reduced ROS accumulation, mitochondrial damage, apoptosis, autophagy-associated responses, and renal injury, with involvement of SIRT5/FOXO3a signalling.	*SIRT5* knockdown worsened toxicity, whereas overexpression was protective. Docking and CETSA suggested interaction with SIRT5 but did not establish binding affinity or in vivo target occupancy.	[18]
Mitochondrial homeostasis and mitophagy-associated signalling	Oleic acid-challenged HepG2 cells	Parent sesamin	Sesamin reduced lipid accumulation, restored autophagy-associated activity, improved mitochondrial homeostasis, and increased PINK1/Parkin-associated markers.	Autophagy inhibition attenuated the lipid-lowering effect, supporting autophagy involvement; PINK1/Parkin dependency and mitophagic flux were not directly demonstrated.	[17]
Mitochondrial and lysosomal quality control	L02 cells exposed to 500 μM elaidic acid (9-*trans*-C18:1)	Parent sesamin, 32 μM	Sesamin reduced ROS and MDA, increased GSH, partially restored ATP and mitochondrial membrane potential, improved mitochondrial morphology, increased autolysosomes, PINK1, Parkin, LC3-II, TFEB nuclear localisation, and LAMP1, and reduced p62.	Eltrombopag and MHY1485 reversed the lipid-lowering effect, supporting TFEB-regulated autophagy–lysosome signalling. The inhibitors are not pathway-specific, mitophagic flux was not directly measured, and evidence was limited to one in vitro model.	[42]
Autophagic flux and lysosomal biogenesis	Human cell cultures expressing tandem mRFP–GFP–LC3	Sesamin, episesamin, and their monocatechol metabolites	Parent lignans and metabolites increased autophagic flux, with stronger effects from monocatechol metabolites. Sesamin and its monocatechol metabolite inhibited mTORC1, reduced ULK1 and TFEB phosphorylation, and promoted isolation-membrane formation and lysosomal biogenesis.	Tandem-reporter data provide comparatively strong evidence of completed autophagic flux and relative mTORC1 selectivity. Organ-specific flux, intracellular exposure, and therapeutic efficacy in vivo were not established.	[14]
Mitochondrial danger signalling	Palmitic acid-exposed USMCs and pregnant mice fed an HFD	Parent sesamin	Sesamin reduced mitochondrial damage, ROS, mtDNA leakage, cGAS–STING activation, IL-1β and IL-18, while improving respiration, glucose uptake, and insulin signalling. In mice, systemic inflammation decreased and uterine insulin sensitivity improved.	mtDNA depletion and STING knockdown attenuated inflammatory and metabolic responses, supporting upstream mitochondrial involvement. Docking did not establish direct STING binding, and findings were not confirmed in human uterine tissue.	[19]

Abbreviations: AKI, acute kidney injury; ARE, antioxidant response element; ATP, adenosine triphosphate; CETSA, cellular thermal shift assay; cGAS, cyclic GMP–AMP synthase; CNS, central nervous system; EC1, episesamin-derived monocatechol metabolite; GFP, green fluorescent protein; GSH, reduced glutathione; HFD, high-fat diet; HO-1, heme oxygenase-1; IL, interleukin; LAMP1, lysosome-associated membrane protein 1; LC3, microtubule-associated protein 1 light chain 3; MDA, malondialdehyde; mRFP, monomeric red fluorescent protein; mtDNA, mitochondrial DNA; mTORC1, mechanistic target of rapamycin complex 1; NRF2, nuclear factor erythroid 2-related factor 2; p38 MAPK, p38 mitogen-activated protein kinase; ROS, reactive oxygen species; SC1, sesamin-derived monocatechol metabolite; STING, stimulator of interferon genes; TFEB, transcription factor EB; ULK1, Unc-51-like autophagy-activating kinase 1; USMC, uterine smooth muscle cell.

**Table 3:** Experimental evidence linking sesamin to regulated cell death in non-malignant and malignant models.

RCD Pathway/Direction	Model/Stressor	Sesamin Exposure	Key Findings	Validation/Limitation	Ref.
Ferroptosis suppression; cytoprotection	Perfluorooctanesulfonate-induced liver injury	Parent sesamin in vivo	Sesamin reduced ferroptosis-related biochemical and molecular changes in the liver.	Hepatocyte-specific *Foxo1* deletion abolished protection, providing strong evidence of FOXO1 dependency; direct targeting and human exposure relevance were not established.	[21]
Ferroptosis suppression linked to autophagy; cytoprotection	APAP-challenged C57BL/6J mice; APAP-exposed AML12 and HepG2 cells	Mice: 50 or 100 mg/kg/day for 4 weeks before APAP 300 mg/kg; cells: 40 μM sesamin with 20 mM APAP	Sesamin improved viability, enhanced autophagy-associated responses, and reduced ferroptosis-related liver injury.	Fer-1 (2 μM) improved viability, whereas 3-MA (5 mM) abolished sesamin-mediated protection. Hepatocyte-specific *Foxo1* deletion eliminated autophagy enhancement and ferroptosis suppression. Direct FOXO1 targeting was not established.	[44]
Ferroptosis suppression; vascular protection	Apolipoprotein E-deficient mice; oxidised LDL-exposed HUVECs	Mice: 50 or 100 mg/kg/day for 12 weeks; cells: 50 μM sesamin for 2 h before 100 μg/mL oxidised LDL	Sesamin reduced vascular injury, ferrous iron, lipid oxidation, and MDA; increased GSH/GSSG; and shifted ACSL4, GPX4, and SLC7A11 in an anti-ferroptotic direction.	Fer-1 (1 μM) produced a similar phenotype. *SREBF1* silencing weakened protection, whereas concurrent *METTL3* knockdown partially restored it. Intracellular sesamin/metabolite exposure was not measured.	[22]
Ferroptosis suppression; ischaemic tissue protection	Rat random-pattern skin-flap model; HUVECs subjected to oxygen–glucose deprivation/reoxygenation	Parent sesamin in vivo and in vitro	Sesamin increased flap survival and was associated with NRF2/SLC7A11/GPX4 activation.	ML385 weakened sesamin-induced protection; Fer-1 served as a positive ferroptosis control. Evidence was pharmacological; genetic confirmation and exposure measurements were lacking.	[23]
Ferroptosis- and apoptosis-related suppression; renal cytoprotection	APAP-exposed HK-2 cells and APAP-induced nephrotoxicity	Cells: 40 μM sesamin with 20 mM APAP; hemin 50 μM; ZnPP IX 10 μM	Sesamin restored *HMOX1* expression and attenuated apoptosis- and ferroptosis-related changes.	Hemin reproduced and ZnPP IX substantially abolished protection, supporting an HO-1-dependent component. This does not establish a universal anti-ferroptotic role for HO-1.	[45]
Pyroptosis suppression; hepatoprotection	HFHCD-induced NASH in mice; palmitate-exposed primary mouse hepatocytes	Mice: 120 mg/kg/day for 6 weeks (*n* = 10/group); hepatocytes: 50 or 100 μM sesamin with 0.5 mM palmitate for 48 h	Sesamin reduced NLRC4, cleaved caspase-1, GSDMD-N, mature IL-1β, and LDH release.	PMA (200 nM) increased PKCδ phosphorylation and attenuated suppression of the NLRC4–caspase-1–GSDMD axis. PMA is not PKCδ-selective, and genetic inflammasome/gasdermin validation was lacking.	[20]
Pyroptosis- and apoptosis-associated cell death; antitumour activity	Murine EL4 T-cell lymphoma cells and mouse lymphoma model	Cells: 10, 20, or 40 μM sesamin for 72 h; parent sesamin also administered in vivo	Autophagy preceded apoptosis- and pyroptosis-associated changes; sesamin also reduced tumour growth.	Autophagy inhibition diminished both death phenotypes, supporting a contributory role for autophagy-associated processes. Pharmacological inhibition alone does not establish autophagy as the executing death mechanism.	[25]
Apoptosis suppression; renal cytoprotection	Diquat-exposed HK-2 cells and mice with acute kidney injury	Parent sesamin in vitro and in vivo	Sesamin reduced oxidative stress, mitochondrial damage, apoptosis, autophagy-associated responses, and renal injury, with involvement of SIRT5/FOXO3a signalling.	*SIRT5* knockdown worsened toxicity and overexpression was protective. Apoptosis suppression was not shown to be independent of the broader reduction in oxidative injury.	[18]
Apoptosis induction; antitumour activity	HeLa and SiHa cervical cancer cells; normal dermal fibroblasts for comparison	Sesamin 75 or 150 μM for 24 h; pifithrin-α 30 μM before 150 μM sesamin	At 150 μM, sesamin increased sub-G1 and annexin V-positive fractions, increased p53 phosphorylation, PUMA, BAX, and PTEN, and reduced AKT phosphorylation.	Pifithrin-α reduced apoptosis and restored proliferation, supporting p53 involvement. Direct p53 activation was not established, and concentrations greatly exceeded reported human parent-sesamin exposure.	[26]
Apoptosis-associated chemosensitisation	A549 and CL1-5 lung adenocarcinoma cells treated with taxanes	Sesamin 100 μM with docetaxel 12.5 μM or paclitaxel 5 μM	Combinations reduced viability and colony formation, produced combination indices < 1, and increased caspase-3 and PARP cleavage.	No caspase-inhibitor rescue was reported; findings support chemosensitisation accompanied by apoptotic signalling rather than exclusive apoptotic execution.	[27]
ROS- and autophagy-associated apoptosis; antitumour activity	Nasopharyngeal carcinoma cells and BALB/c nude-mouse xenografts	Cells: 20 or 50 μM sesamin; NAC 5 mM or 3-MA 3 mM before 50 μM sesamin; mice: 50 or 100 mg/kg/day for 14 days (*n* = 6/group)	Sesamin reduced proliferation and mitochondrial membrane potential, increased ROS and apoptosis-associated proteins, and altered autophagy markers. In mice, tumour volume and weight decreased and cleaved caspase-3 increased.	NAC and 3-MA attenuated cellular effects, supporting contributions from oxidative signalling and autophagy. Limited inhibitor specificity and absence of an autophagic-flux assay preclude definitive causal ordering.	[46]

Abbreviations: 3-MA, 3-methyladenine; ACSL4, acyl-CoA synthetase long-chain family member 4; AKT, protein kinase B; APAP, acetaminophen; BAX, BCL-2-associated X protein; Fer-1, ferrostatin-1; FOXO, forkhead box O; GPX4, glutathione peroxidase 4; GSDMD-N, N-terminal fragment of gasdermin D; GSH/GSSG, reduced-to-oxidised glutathione ratio; HFHCD, high-fat, high-cholesterol diet; HO-1, heme oxygenase-1; HUVEC, human umbilical vein endothelial cell; IL-1β, interleukin-1β; LDH, lactate dehydrogenase; MDA, malondialdehyde; METTL3, methyltransferase-like 3; NASH, nonalcoholic steatohepatitis; NLRC4, NLR family CARD domain-containing 4; NRF2, nuclear factor erythroid 2-related factor 2; PARP, poly(ADP-ribose) polymerase; PKCδ, protein kinase Cδ; PMA, phorbol 12-myristate 13-acetate; PTEN, phosphatase and tensin homologue; PUMA, p53 upregulated modulator of apoptosis; RCD, regulated cell death; ROS, reactive oxygen species; SIRT5, sirtuin 5; SLC7A11, solute carrier family 7 member 11; SREBF1, sterol regulatory element-binding transcription factor 1; ZnPP IX, zinc protoporphyrin IX.

**Table 4:** Human intervention studies and pooled clinical evidence for sesamin-related preparations.

Population/Design	Intervention	Key Findings	Critical Interpretation	Ref.
Healthy adults; single-blind, randomised, placebo-controlled, parallel-group study (*n* = 48; active *n* = 24; PK subset *n* = 10)	Sesamin/episesamin preparation (1:1); 50 mg/day for 28 days	Parent sesamin and episesamin reached steady state by day 7 without detectable accumulation. No serious adverse events were reported.	Principal short-term human exposure and tolerability study, but based on a mixed-lignan preparation, small PK subset, and no tissue-exposure or efficacy assessment.	[15]
Middle-aged adults with mild hypertension; double-blind, placebo-controlled crossover study (*n* = 25)	Sesamin/episesamin mixture (~1:1); 60 mg/day for 4 weeks per period with a 4-week washout	During the active period, systolic blood pressure decreased from 137.6 ± 2.2 to 134.1 ± 1.7 mmHg and diastolic pressure from 87.7 ± 1.3 to 85.8 ± 1.0 mmHg (*p* = 0.044 and 0.045, respectively).	Results were reported as within-period changes; a treatment-by-period effect estimate was not provided. Sample size and duration were limited.	[50]
Adults with type 2 diabetes; randomised, double-blind, placebo-controlled trial (*n* = 48)	Sesamin 200 mg/day or placebo for 8 weeks	Sesamin reduced fasting blood glucose, HbA1c, and TNF-α versus placebo (all *p* < 0.05). Other anthropometric and inflammatory changes were mainly within-group findings.	Small, short-duration trial without assessment of diabetic complications, durability, or clinical outcomes.	[51]
Women with rheumatoid arthritis; placebo-controlled trial (*n* = 44), reported in two publications	Sesamin 200 mg/day or placebo for 6 weeks	Between-group differences were reported for MDA, total antioxidant capacity, and HDL-C [52]; a second report described reductions in high-sensitivity C-reactive protein and TNF-α and changes in selected matrix and clinical measures [53].	Both publications analysed the same cohort and should be considered one trial. Several outcomes changed only within the sesamin group.	[52,53]
Participants with low-arousal-threshold obstructive sleep apnoea or at risk of obstructive sleep apnoea; double-blind self-controlled crossover study (*n* = 33 enrolled; 25 completed; 20 analysed)	Sesamin 94.0 mg/day; two 8-week periods separated by a 2-week washout	Sleep-quality and sleepiness scores did not differ significantly from placebo. Plasma GPX showed a significant between-period difference (*p* = 0.0215), whereas other redox biomarkers did not.	The isolated GPX finding was not accompanied by consistent biomarker changes or placebo-controlled improvement in subjective sleep outcomes; attrition reduced the analysed sample.	[54]
Systematic review and meta-analysis of seven randomised trials (*n* = 212)	Sesamin-related interventions for 28–60 days	Pooled analyses showed reductions in TC, LDL-C, and systolic blood pressure; no significant pooled effects were found for HDL-C, triglycerides, diastolic blood pressure, or body weight.	Evidence was limited by small trial numbers, short durations, and heterogeneous preparations; pooled effects cannot be attributed exclusively to chemically defined parent sesamin.	[55]

Abbreviations: GPX, glutathione peroxidase; HbA1c, haemoglobin A1c; HDL-C, high-density lipoprotein cholesterol; LDL-C, low-density lipoprotein cholesterol; MDA, malondialdehyde; PK, pharmacokinetic; TC, total cholesterol; TNF-α, tumour necrosis factor-α.

## Data Availability

No new data were created or analyzed in this study. Data sharing is not applicable to this article.
